# Pan‐Cancer Analyses of Necroptosis, Pyroptosis and Ferroptosis Related Genes Reveal TLR4 as A Potential Therapeutic Target

**DOI:** 10.1111/jcmm.70742

**Published:** 2025-07-22

**Authors:** Yu Shen, Yishu Deng, Yali Xie, Tao Zeng, Yongjing Liu, Junwan Lu, Jinyan Huang

**Affiliations:** ^1^ Biomedical Big Data Center, the First Affiliated Hospital Zhejiang University School of Medicine Zhejiang China; ^2^ Center of Regenerative Medicine, Zhejiang University School of Medicine Zhejiang University‐University of Edinburgh Lnstitute Zhejiang China; ^3^ Medical Molecular Biology Laboratory, School of Medicine Jinhua University of Vocational Technology Jinhua China; ^4^ Zhejiang Provincial Key Laboratory of Pancreatic Disease, the First Affiliated Hospital Zhejiang University School of Medicine Zhejiang China; ^5^ Zhejiang University Cancer Center Zhejiang University Zhejiang China

**Keywords:** ferroptosis, necroptosis, pan‐cancer, pyroptosis, TLR4

## Abstract

Programmed cell death is essential for maintaining cellular homeostasis, and emerging forms such as necroptosis, pyroptosis, and ferroptosis (NPF) are associated with cancer progression. However, their exact roles remain poorly characterised. In this study, we investigated the role of NPF‐related genes in cancer prognosis by analysing gene expression and clinical data from the TCGA pan‐cancer dataset. A multigene prognostic model was developed to predict patient survival. Pathway enrichment and tumour microenvironment analyses revealed significant associations between NPF‐related genes and immune cell types, particularly highlighting the link between *TLR4* expression and macrophage infiltration. Elevated *TLR4* expression in tumour cells was correlated with an immunosuppressive environment, positioning *TLR4* as a potential therapeutic target. Moreover, treatment with the *TLR4* inhibitor TAK‐242 was shown to inhibit cell proliferation and migration in PANC‐1 and SW1990 cell lines. These findings underscore the potential of NPF‐related pathways as prognostic biomarkers and support TAK‐242 as a promising therapeutic candidate for cancer treatment.

## 1 Introduction

Regulated cell death (RCD) is a fundamental biological process that ensures tissue homeostasis and proper development by eliminating damaged or superfluous cells in a controlled manner [1]. Among the various types of RCD, apoptosis is the most extensively studied and is traditionally known as the primary form of programmed cell death [2]. However, accumulating evidence has identified additional RCD forms—such as necroptosis, pyroptosis, and ferroptosis—that are mechanistically distinct from apoptosis and play critical roles in cancer. These non‐apoptotic cell death pathways not only bypass defective apoptosis but are also closely associated with immune responses [3]. Despite this, their precise role in tumorigenesis remains to be fully understood. Given the global burden of cancer and the growing body of pan‐cancer research, understanding these pathways is essential for improving patient outcomes [4, 5].

Necroptosis is a caspase‐independent, regulated necrotic process mediated by RIPK1 and RIPK3, which activate MLKL to disrupt cell membrane integrity and trigger cell death [6, 7]. Pyroptosis, an inflammatory form of cell death, is induced by caspase‐1/4/5/11 through inflammasome activation and gasdermin‐mediated pore formation [8, 9, 10]. Ferroptosis is driven by iron‐dependent lipid peroxidation and ROS accumulation, often caused by GPX4 inhibition or iron overload [11, 12, 13]. These three forms of non‐apoptotic regulated cell death play a dual role in tumour progression [7, 14, 15, 16, 17, 18]. Pyroptosis releases pro‐inflammatory cytokines like IL‐18, which recruit immune cells and promote tumour growth [19]. Conversely, tumour‐specific *GPX4* degradation enhances ferroptosis, boosting the anti‐tumour immune response in pancreatic cancer [20]. These findings underscore the complex interplay of RCD mechanisms in cancer and highlight their therapeutic potential.

Necroptosis, pyroptosis, and ferroptosis (NPF) related genes regulate the crosstalk between cell death pathways and tumour progression, impacting immune infiltration in the tumour microenvironment (TME) [21, 22]. This regulation holds potential as a prognostic marker and may influence the effectiveness of immunotherapy [23, 24]. Given the intricate interactions among these pathways, a comprehensive analysis of NPF‐related genes may provide a more accurate assessment of tumour progression and patient prognosis. Moreover, given the heterogeneous roles of NPF‐related genes across different cancer types, a pan‐cancer analysis enables the identification of both shared and cancer‐specific prognostic signatures, which could facilitate more personalised therapeutic strategies.

This study aims to provide a comprehensive analysis of NPF‐related genes across cancer types and construct a robust prognostic model and uncover potential therapeutic vulnerabilities that could inform future precision oncology interventions. By integrating gene expression profiles and clinical outcomes, we assessed NPF pathways activity (NPF score) using ssGSEA. Subsequently, based on LASSO‐Cox regression, we constructed a prognostic model to stratify patients based on risk score. Furthermore, we investigated the therapeutic potential of TAK‐242, a TLR4‐specific inhibitor, through in vitro experiments. This research advances our understanding of NPF pathways in cancer and highlights TLR4 as a promising target for therapeutic intervention.

## 2 Materials and Methods

### 2.1 Collecting Necroptosis, Pyroptosis and Ferroptosis Related Genes

To compile a comprehensive list of necroptosis, pyroptosis, and ferroptosis related genes, we utilised the Molecular Signatures Database (MSigDB). Specifically, we accessed the ‘C5 Ontology Gene set’ module and the ‘C2 Canonical Pathways subcollections’ in MSigDB, sequentially retrieving gene sets associated with necroptosis, pyroptosis, and ferroptosis. In addition, we searched PubMed to identify additional genes relevant to these processes, resulting in a total of 167 genes. The complete gene list and related references are provided in Table S1.

### 2.2 Data Collection and Processing

Gene expression and clinical data were obtained from TCGA via the UCSC Xena platform, including RNA‐seq data from 10,497 samples across 33 cancer types. Normal tissue data from 31 tissue types were downloaded from the GTEx database. To ensure consistency, we retained 23 cancer types with sufficient normal comparison in both TCGA and GTEx (Table S2). Samples with missing clinical information were excluded. Expression data were normalised to TPM, and batch effects were corrected using the removeBatchEffect from the limma package. Additionally, the GSE71729 dataset (145 pancreatic tumours and 46 normal samples) was downloaded for external validation.

### 2.3 Differential Expression and Correlation Analysis

Differential expression analysis of NPF related genes across 23 cancer types was performed using the DESeq2 package in R [25] with variance stabilising transformation (VST) applied to reduce batch effects. Additionally, the STRING database was used to retrieve interactions between NPF related genes and their corresponding proteins [26].

### 2.4 Prognostic Analysis of NPF Related Genes

Clinical data and NPF gene expression profiles from TCGA were used to assess their association with patient prognosis via univariate Cox regression, performed using the survival package in R. Hazard ratios and corresponding *p*‐values were calculated, and results were visualised in a heatmap generated with the pheatmap package. Genes identified as risk factors in tumours are highlighted in red, and protective factors are highlighted in blue (*p* < 0.05).

### 2.5 Calculation of NPF Score

Single‐sample gene set enrichment analysis (ssGSEA) was performed on 10,497 samples to calculate activity scores for necroptosis, pyroptosis, and ferroptosis based on the expression of NPF related genes [27]. The NPF scores for 23 cancer types were then ranked in ascending order and visualised as boxplots using the ‘ggplot2’ package. Differences in NPF scores between tumour and normal tissues for specific cancer types were analysed and visualised using the ‘ggpubr’ package, based on batch‐corrected expression matrices.

### 2.6 Prognosis Analysis of NPF Score

The association between NPF scores and patient prognosis was assessed using four survival outcomes: overall survival (OS), disease‐specific survival (DSS), disease‐free interval (DFI), and progression‐free interval (PFI). Univariate Cox regression was performed with the survival and survminer packages in R. Results were visualised using base R plotting functions.

### 2.7 Identification of Prognostic Genes

To further investigate the relationship between the expression levels of NPF related genes and OS in cancer patients, we identified differentially expressed genes associated with OS (Table S3). LASSO Cox regression was then applied to eliminate gene collinearity and reduce the number of genes. Finally, multivariate Cox regression analysis was performed to identify independent prognostic genes.

### 2.8 Construction and Validation of a Prognostic Model

The risk score was calculated using centralised and standardised mRNA expression data, as follows: Risk score = Σ (x_i_ × y_i_), i = 1 to n. *X* represents the coefficients of genes derived from LASSO Cox regression analysis, *Y* denotes gene expression levels, and n indicates the total number of genes included in the risk model. Lasso Cox regression analysis was performed using the R package ‘glmnet’ [28], and the optimal lambda was determined using the 1‐SE rule. Patients from all cancer types were stratified into high‐risk and low‐risk groups based on the median risk score. Overall survival was analysed for these groups, stratified by cancer type. Receiver operating characteristic (ROC) curves were generated using the ‘timeROC’ package to assess the prognostic performance of the model [29].

### 2.9 Gene Set Variation Analysis (GSVA)

GSVA was performed to identify and analyse differences in enriched pathways between the high‐risk and low‐risk groups [27]. The analysis utilised gene sets from the MSigDB database, including the ‘C2 CP Gene set’, ‘C5 Ontology Gene set’, and ‘Hallmark genesets’.

### 2.10 Immunologic Infiltration Analysis

The relationship between prognostic genes and related immune cells was investigated based on the original data from the TIMER2 online database. Three algorithms (CIBERSORT, CIBERSORT ‐ABS and TIMER) were used to examine immune cell infiltration. To reduce the impact of tumour purity, only samples with purity > 0.6, as estimated by the ESTIMATE [30], were included in the analysis. Results were visualised using the ggplot2 package in R.

### 2.11 Cell Culture

The PANC‐1 and SW1990 cell lines were purchased from the Shanghai Institute of Biochemistry and Cell Biology (Shanghai, China). Dulbecco's modified Eagle's medium (DMEM, Gibco, Life Technologies, Grand Island, NY, USA) was used for cell culture, in which 1% penicillin–streptomycin was added (100 U/mL and 100 mg/mL; Beyotime Biotechnology) and 10% fetal bovine serum (FBS, Life Technologies, Grand Island, NY, USA). Cells were cultured in a constant‐temperature incubator at 37°C under 5% CO2 and 95% air under normoxic conditions. All cell cultures were tested negative for mycoplasma contamination during this study. The following reagents were used in the experiments: Resatrovid (TAK‐242; MCE, HY‐11109) and RSL3 (MCE, HY‐100218A).

### 2.12 Cell Viability Assay

Cells were cultured in 96‐well plates and treated with TAK‐242 or with DMSO as a control, for 0, 12, 24, 36, and 48 h. At the end of the treatment, 10 μL of cell counting kit‐8 (CCK‐8) reagent (Absin, abs50003) was added to each well and incubated for 2 h. At the end of this incubation, the absorbance at 450 nm was measured using a microplate reader (Bio‐Rad, Hercules, CA, USA). Relative cell viability was calculated as the ratio of the absorbance in each treatment group to that of the control group.

### 2.13 Colony Formation Assay

PANC‐1 and SW1990 cells (1 × 10^3^ cells/well) were seeded into 6‐well plates and incubated for 7 days. The medium was then replaced with DMEM containing TAK‐242 or DMSO control, and cells were cultured for an additional 7 days. Colonies were fixed with methanol for 15 min and stained with 0.5% crystal violet for 20 min. After washing with water and air drying, images were captured and analysed using ImageJ Plus software. Each treatment was performed in triplicate.

### 2.14 Cell Migration Assay

Cell migration was evaluated using a 24‐well transwell chamber with an 8.0 μm pore membrane (PIEP12R48, Millipore). PANC‐1 and SW1990 cells (4 × 10^4^ cells/well) were seeded in the upper chamber in serum‐free DMEM. The lower chamber contained DMEM with 10% FBS as a chemoattractant. After 12 h, the upper medium was replaced with serum‐free DMEM containing TAK‐242 or DMSO, and cells were incubated for another 36 h. Migrated cells were fixed with methanol, stained with 5% crystal violet for 20 min, and washed with PBS. Images were taken from three random fields per insert using an inverted microscope and quantified using ImageJ Plus software.

### 2.15 Lipid Peroxidation Assay

Cells were cultured and treated with the indicated reagents. Following treatment, cells were harvested and resuspended in 1 mL of DMEM containing 10% bovine calf serum (BCS) and 10 μM C11‐BODIPY581/591 (Thermo Fisher Scientific, Cat. #D3861). The samples were incubated at 37°C for 30 min. After incubation, cells were washed with PBS and analysed by flow cytometry (Fortessa, BD Biosciences).

### 2.16 Ferroptosis Susceptibility Assay

Cells were treated with TAK‐242 or DMSO for 72 h. After treatment, equal numbers of cells were harvested and exposed to ferroptosis inducers RSL3 for 24 h. Following induction, cells were stained with Annexin V and DAPI according to the manufacturer's instructions to assess cell viability. Samples were analysed by flow cytometry (Fortessa, BD Biosciences) to evaluate the proportion of viable and non‐viable cells.

### 2.17 RNA‐Seq

PANC‐1 cells were treated with 40 μM TAK‐242 or DMSO for 72 h. Total RNA was extracted using the RNeasy Mini Kit (Qiagen). High‐quality RNA (RIN > 7) was used for library preparation. mRNA was enriched using poly‐T oligo‐attached magnetic beads, fragmented, and reverse‐transcribed into cDNA using random hexamer primers. After second‐strand synthesis, end repair, A‐tailing, adaptor ligation, size selection, and PCR amplification, libraries were sequenced on the Illumina NovaSeq 6000 platform with 2 × 100 bp paired‐end reads.

Pair‐end sequencing FASTQ files were aligned to the human reference genome (hg38). Raw gene counts were derived from the read alignments by Featurecounts [31]. Differential expression analysis was performed using DESeq2 [25]. GO enrichment analysis and GSEA were used for pathway analysis.

### 2.18 Statistical Analysis

All statistical data in this study were performed in at least three independent experiments. Data between the two groups were compared with the student t‐test. All statistical analyses were performed by GraphPad Prism 7.0 Plus (GraphPad Software, La Jolla, CA, USA). A *p*‐value of less than 0.05 was considered statistically significant (* for *p* < 0.05, ** for *p* < 0.01, *** for *p* < 0.001; ns = not significant).

## 3 Result

### 3.1 Expression and Correlation Analysis of NPF Related Genes Across Tumour Types

**FIGURE 1:**
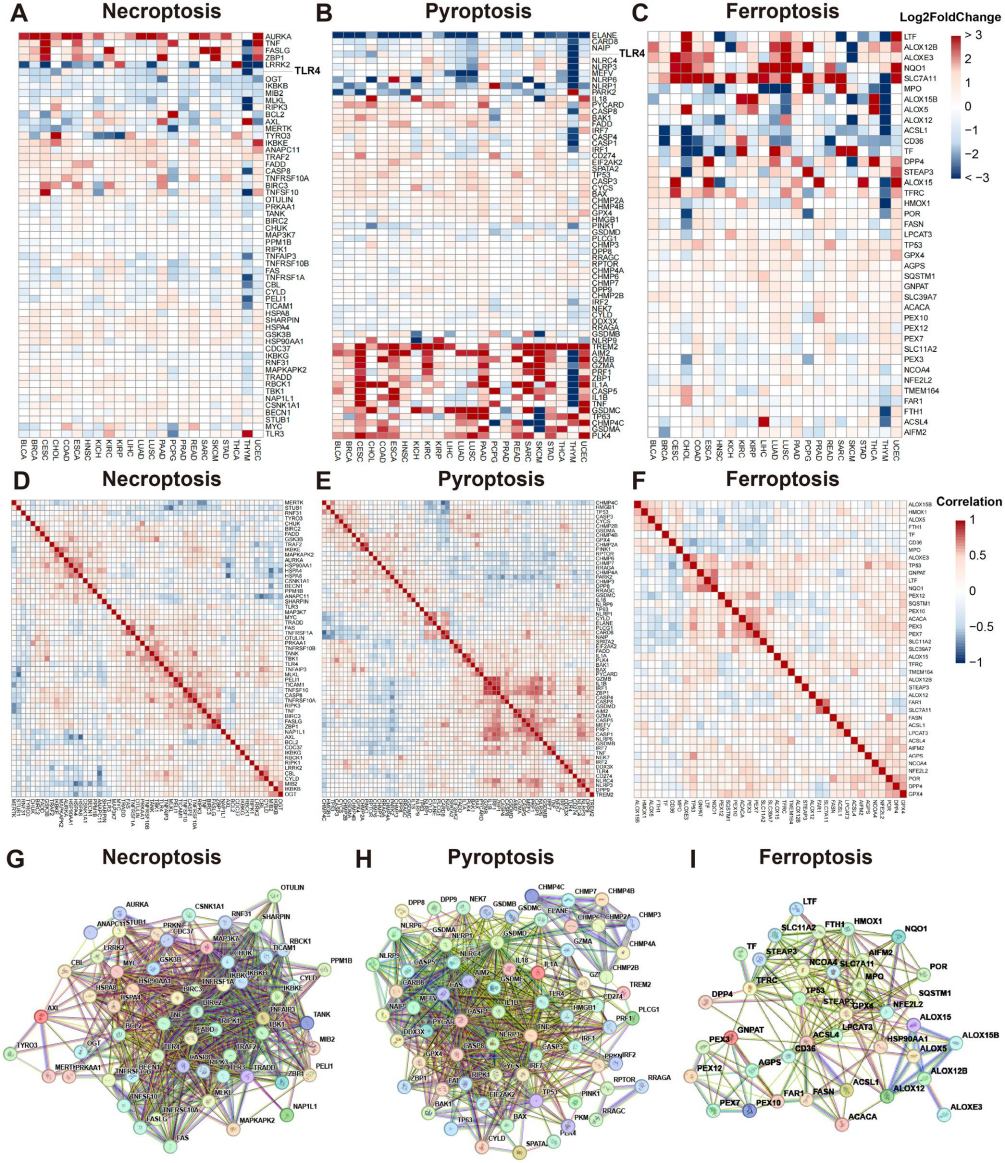
Expression of NPF related genes in different tumours and their correlations. (A–C) Differential expression analysis of NPF related genes. The colour scale represents log_2_foldchange values, with red indicating upregulation (log_2_FoldChange > 0, *p*‐value < 0.05) and blue indicating downregulation (log_2_FoldChange < 0, *p*‐value < 0.05); (D–F) Correlation analysis of NPF related genes; (G‐I) Protein–protein interactions of NPF related genes.

To evaluate the expression profiles of NPF related genes across various tumour types, we analysed data from over 10,000 samples in the TCGA and GTEx database. Due to the lack of normal tissue data for certain cancer types in the TCGA database, such as MESO, UVM, and GBM, we focused on comparing 23 types of cancer tissues with their respective normal tissues. The abbreviations for these 23 cancer types can be found in Table S2. As illustrated in Figure 1A–C, the expression of NPF related genes showed significant variability among different tumour types.

We further conducted correlation analyses for genes involved in the necroptosis, pyroptosis, and ferroptosis pathways separately (Figure 1D–F). Each pathway showed distinct patterns of interconnected gene expression, indicating a certain level of correlation among the genes within each pathway. Interestingly, some genes, such as *TLR4*, were involved in multiple pathways (e.g., necroptosis and pyroptosis) and exhibited different correlation relationships depending on the pathway, suggesting potential cross‐pathway interactions.

To explore these interactions further, we conducted protein–protein interaction (PPI) analyses, which revealed significant interactions within each pathway. Moreover, those genes differentially linked by expression among multiple pathways (e.g., TLR4) were also identified to play crucial roles in protein level, underscoring their potential roles in mediating cross‐pathway signalling (Figure 1G–I).

### 3.2 Relationship Between NPF Score and Clinical Characteristics

**FIGURE 2:**
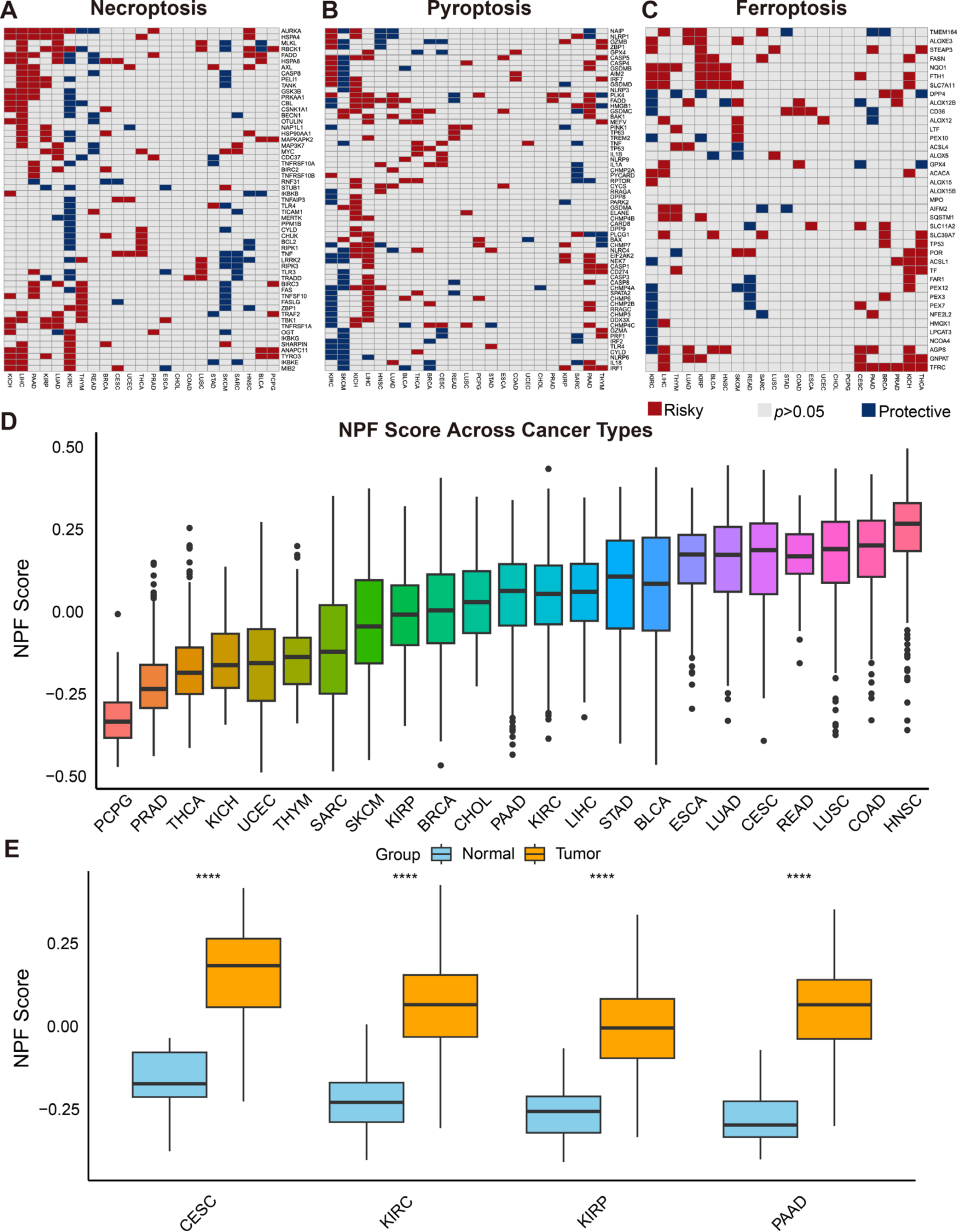
Relationship between the expression of NPF related genes and clinical data. (A–C) Relationship between the expression of necroptosis, pyroptosis and ferroptosis related genes and clinical prognosis in different tumours. Red and blue represent positive and negative correlations, respectively, while grey represents *p*‐value > 0.05; (D) NPF score of different tumours. The size of the NPF score increased from left to right in each of the 23 tumours; (E) Differences in NPF score between tumour and normal tissues for the four cancer types with the most significant *p*‐values: CESC, KIRC, KIRP and PAAD. A two‐sided Wilcoxon signed‐rank test was used for statistical analysis (****p < 0.0001).

To assess whether NPF‐related genes act as independent prognostic factors, we performed univariate Cox regression using clinical data from 23 TCGA cancer types. As illustrated in Figure 2A–C, these genes exhibited diverse prognostic roles across cancers. For instance, *AURKA* was consistently associated with poor prognosis, while NLRP6 appeared protective in most tumour types. To further explore the activity of necroptosis, pyroptosis, and ferroptosis in individual tumour samples, we used ssGSEA to score the overall activity of these pathways. We then ranked the NPF scores of 23 cancer types from low to high (Figure 2D), identifying PCPG and HNSC as having the lowest and highest NPF scores. Comparison of NPF scores between tumour and normal tissues showed significant differences in 19 of the 23 cancer types analysed (*p* < 0.05; Figure 2E and Figure S1).

Next, we conducted a univariate Cox analysis using the NPF score combined with clinical data to assess its prognostic value. Four indices were used to evaluate correlations between NPF scores and patient prognosis: OS, DSS, DFI, and PFI. Cancer types were arranged in ascending order based on *p*‐values.

**FIGURE 3:**
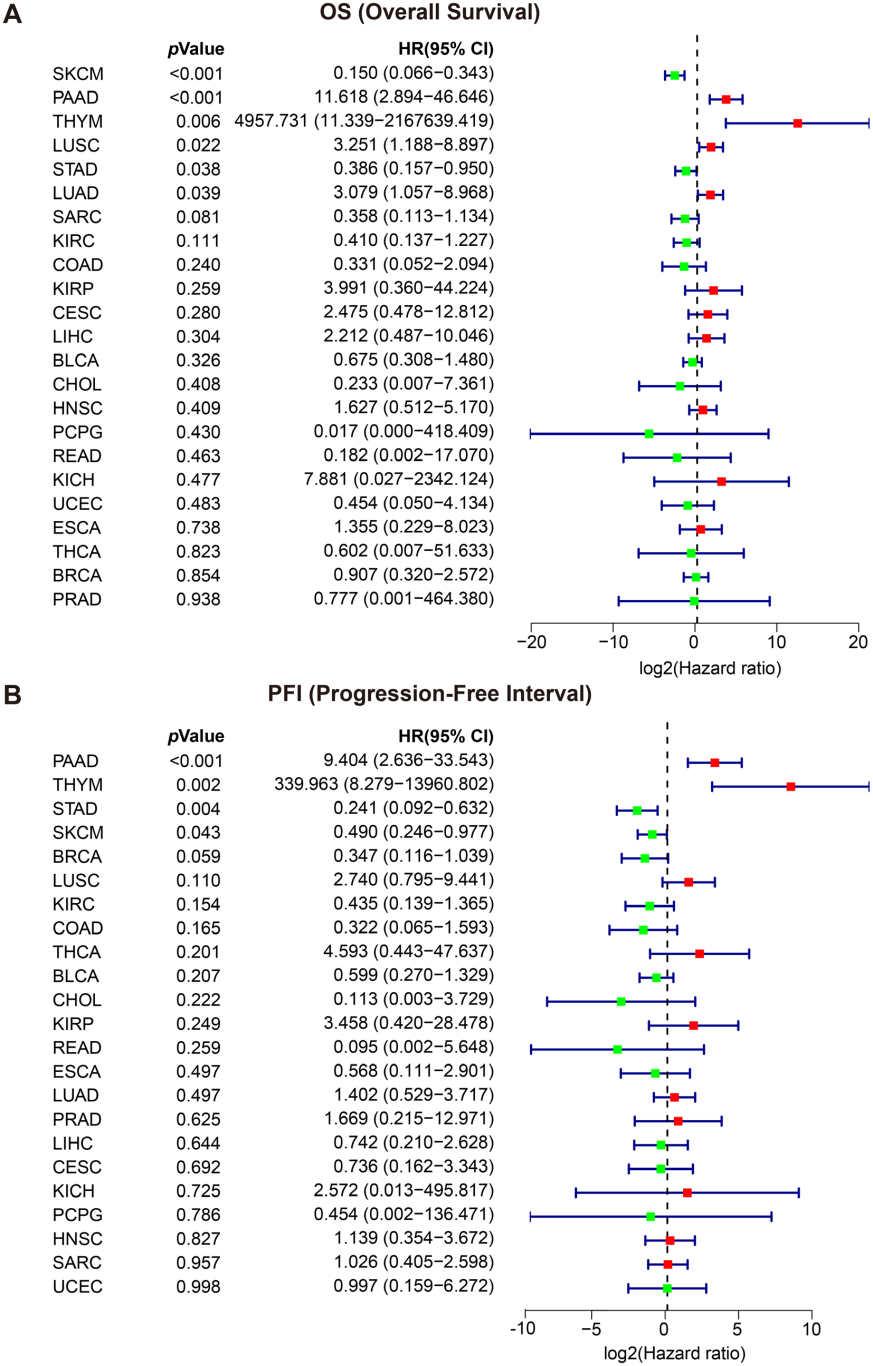
Relationship between the NPF score and clinical data. (A) Hazard ratio showing that NPF score is associated with OS in several tumour types; (B) NPF score is associated with PFI in several tumour types. A two‐sided, univariate Cox proportional hazards regression analysis was used. HRs with 95% confidence intervals (CIs) are plotted for each tumour type. The colour of the forest plot was annotated according to the HR. Red represents NPF score as a risk factor in the indicated cancer type, while Green represents NPF score as a protective factor. Sample sizes: BLCA (*n* = 407), BRCA (*n* = 1098), CESC (*n* = 306), CHOL (*n* = 36), COAD (*n* = 288), ESCA (*n* = 182), HNSC (*n* = 520), KICH (*n* = 66), KIRC (*n* = 531), KIRP (*n* = 289), LIHC (*n* = 371), LUAD (*n* = 515), LUSC (*n* = 498), PAAD (*n* = 179), PCPG (*n* = 182), PRAD (*n* = 496), READ (*n* = 92), SARC (*n* = 262), SKCM (*n* = 469), STAD (*n* = 414), THCA (*n* = 512), THYM (*n* = 119), UCEC (*n* = 181).

As presented in Figure 3A, the OS of six cancer types was significantly associated with NPF scores. Among these, PAAD, THYM, LUSC, and LUAD were identified as risk factors, whereas SKCM (*p* < 0.001) and STAD (*p* < 0.001) were protective factors. For DSS, the NPF score was also a risk factor for PAAD and THYM, while it served as a protective factor for SKCM and STAD (Figure S2A). Additionally, the NPF score was significantly correlated with DFI in three cancer types (Figure S2B) and with PFI in four cancer types (Figure 3B). Overall, these findings indicate that the NPF score is significantly associated with clinical survival, therapeutic efficacy, and recurrence in patients with PAAD, SKCM, and THYM, highlighting its potential as a valuable prognostic biomarker in cancer.

### 3.3 Necroptosis, Pyroptosis, and Ferroptosis Activity as Prognostic Predictors in Cancer

To further elucidate the impact of NPF activity on patient survival, we assessed the prognostic significance of NPF‐related gene expression across different tumour types. Kaplan–Meier survival curve analysis was performed after categorising patients into two groups based on the mean NPF scores. The results indicated that NPF activity was significantly associated with OS in 5 out of the 23 tumour types analysed (Figure S3A–E).

**FIGURE 4:**
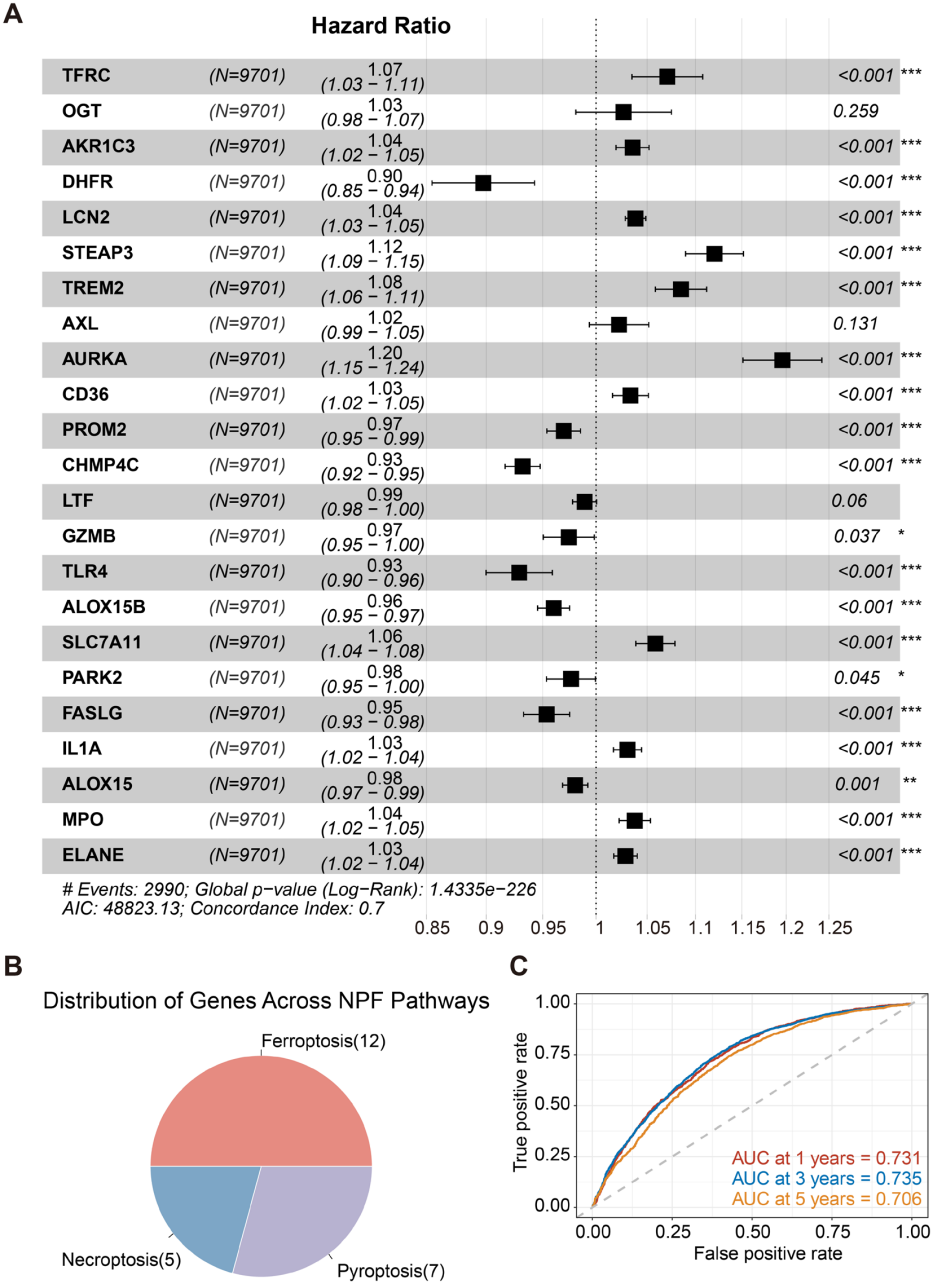
Association of NPF score with prognostic outcomes across various tumour types and pathways. (A) Forest plot showing the hazard ratios and 95% confidence intervals (CI) for genes associated with the NPF score in the cohort (*N* = 9701). A multivariate Cox proportional hazards regression analysis was used to identify significant associations. Genes with significant associations are marked with asterisks (*p < 0.05, **p < 0.01, ***p < 0.001); (B) Pie chart depicting the distribution of genes across NPF pathways; (C) Time‐dependent ROC curves evaluating the predictive efficiency of the NPF risk prognostic model for OS at 1, 3, and 5 years.

To achieve more accurate survival prediction for pan‐cancer patients, we identified NPF genes significantly associated with OS and exhibited differential expressions, which are listed in Table S3. Specifically, the genes shown in Figure 1A–C demonstrated significant differential expression in at least half of the cancer types. Subsequently, we applied LASSO regression followed by multivariate Cox regression analysis on these genes (Figure S3F,G, 4A). The distribution of genes across NPF pathways shows that 12 genes are involved in ferroptosis, 7 in pyroptosis, and 5 in necroptosis (Figure 4B). Using the results of the multivariate Cox regression, we constructed a prognostic model to predict patient survival. Time‐dependent ROC analysis demonstrated that the model's prognostic accuracy for OS was 0.731 at 1 year, 0.735 at 3 years, and 0.706 at 5 years (Figure 4C).

**FIGURE 5:**
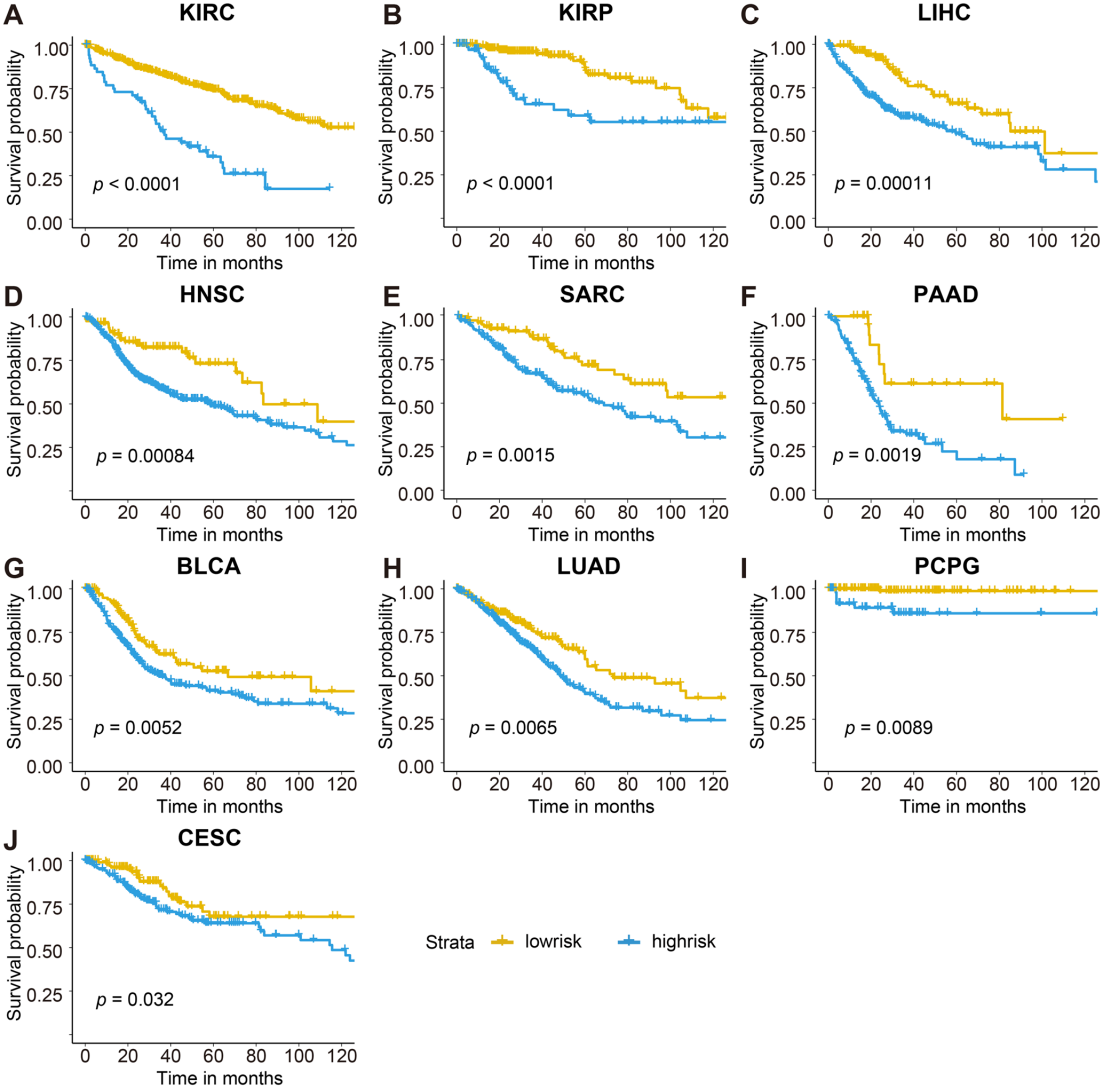
Survival curves for the prognostic signature across 10 tumour types with significant *p*‐value. (A, B) Tumour types with highly significant differences (*p* < 0.0001) in survival probability between the two groups; (C–J) Tumour types with significant differences (*p* < 0.05). A log‐rank test was used for statistical analysis. Sample sizes: KIRC: Low‐risk group (*n* = 476), High‐risk group (*n* = 55); KIRP: Low‐risk group (*n* = 217), High‐risk group (*n* = 69); LIHC: Low‐risk group (*n* = 133), High‐risk group (*n* = 237); HNSC: Low‐risk group (*n* = 83), High‐risk group (*n* = 436); SARC: Low‐risk group (*n* = 86), High‐risk group (*n* = 176); PAAD: Low‐risk group (*n* = 26), High‐risk group (*n* = 153); BLCA: Low‐risk group (*n* = 125), High‐risk group (*n* = 281); LUAD: Low‐risk group (*n* = 181), High‐risk group (*n* = 315); PCPG: Low‐risk group (*n* = 129), High‐risk group (*n* = 53); CESC: Low‐risk group (*n* = 130), High‐risk group (*n* = 176).

Patients were further stratified into high‐ and low‐risk groups according to the mean NPF risk score across all cancer types. Kaplan–Meier survival curves were generated for each cancer type to evaluate the prognostic value of NPF activity within specific tumour types. Notably, significant survival differences between high‐ and low‐risk groups were observed in 10 out of the 23 selected tumour types with at least 10 patients in each group (Figure 5A–J).

### 3.4 Differences in Tumour Microenvironment Immune Cell Composition Associated With *TLR4* Expression and Risk Stratification

To identify potential therapeutic targets, we focused our analysis on PAAD (pancreatic adenocarcinoma) due to its unique characteristics observed in the pan‐cancer analysis. Specifically, PAAD was selected because NPF activity in this cancer type showed significant correlations with various clinical data, including patient outcomes. Furthermore, there were striking differences in overall survival between the high‐ and low‐risk groups in PAAD. Based on these findings, we sought to explore potential therapeutic targets by analysing differences in pathway enrichment between the high‐ and low‐risk groups. Using GSVA, we observed significant enrichment differences in pathways related to cell proliferation, cell cycle regulation, immune response, and inflammation, with notable involvement of the TOLL‐LIKE_RECEPTOR signalling pathway (Figure S4). The TOLL‐LIKE_RECEPTOR signalling pathway, encompassing downstream effectors such as the *TLR4‐IRF3‐7* axis, demonstrated enhanced activity within the high‐risk cohort. This observation underscores its potential as a pivotal therapeutic target for cancer patients, consistent with previous findings on its role in tumour progression and therapeutic responsiveness [32].

**FIGURE 6:**
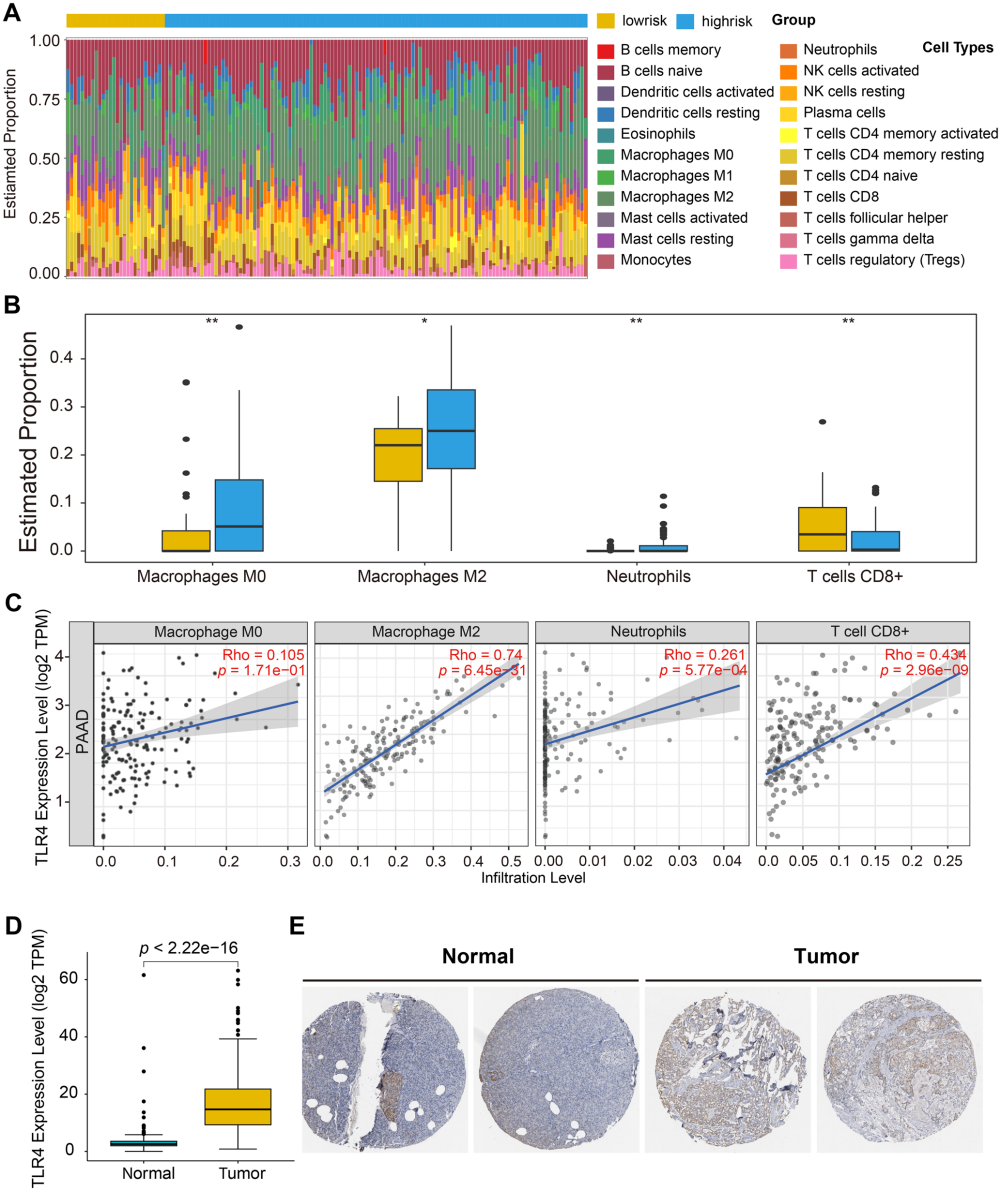
Correlation of NPF risk score and TLR4 expression with TME cell infiltration in PAAD patients. (A) Relative proportion of immune cell infiltration in high‐risk and low‐risk group; (B) Comparison of the proportion of different cell types between lowrisk (*n* = 19) and highrisk (*n* = 129) PAAD samples. A two‐sided Wilcoxon signed‐rank test was performed and only features with *p* < 0.05 are displayed; (C) The relationship of TLR4 expression and different immune cell infiltration levels in PAAD; (D) Boxplot showing the expression levels of TLR4 in normal tissue(*n* = 171) and PAAD(*n* = 179). A two‐sided Wilcoxon signed‐rank test was used; (E) The expression of TLR4 between normal tissues and PAAD at the translational level.

To further investigate differences in tumour microenvironment, we utilised CIBERSORT to quantify the proportions of various immune cell types. Our analysis demonstrated that high‐risk patients exhibited significantly elevated infiltration levels of M0 macrophages, M2 macrophages, and neutrophils, whereas the proportion of resting CD8+ T cells was lower in the high‐risk group compared to the low‐risk group (Figure 6A,B). These findings suggest that these immune cell populations are associated with the risk stratification of cancer patients.

We further analysed the correlation between the expression levels of NPF related genes and the proportion of immune cell infiltration using TIMER (Figure S5). Among these genes, *TLR4* expression exhibited the strongest positive correlation with the proportion of macrophages, particularly M2 macrophages (Figure 6C and Figure S6A), suggesting a potential association between *TLR4* expression and a tumour microenvironment that promotes tumour progression in high‐risk patients [33].

Additionally, we examined the expression levels of TLR4 between normal pancreatic tissues and pancreatic adenocarcinoma (PAAD) tissues at both the transcriptional and translational levels. Transcriptional levels were assessed in the TCGA database and independently validated in the GEO dataset GSE71729, while translational levels were evaluated through immunohistochemical staining, obtained from the Human Protein Atlas (HPA) database [34]. *TLR4* expression was significantly higher in PAAD tissues compared to normal tissues (Figure 6D,E and Figure S6B). These patterns of elevated expression further underscore the potential role of *TLR4* in modulating immune cell infiltration and shaping the tumour microenvironment composition in PAAD [35].

### 3.5 TAK‐242 Inhibits the Growth of Pancreatic Cancer Cells and Alters Susceptibility to Ferroptosis

**FIGURE 7:**
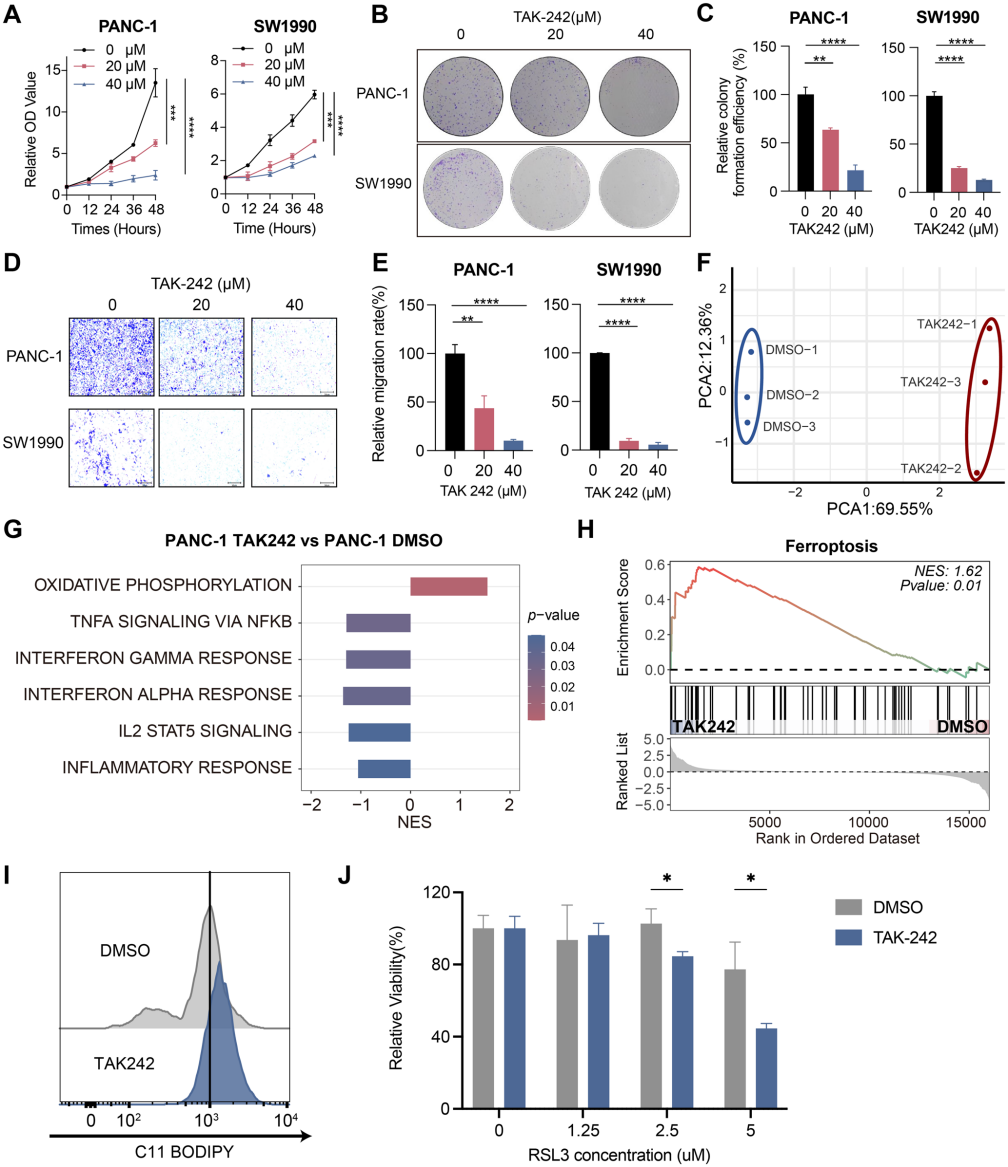
TAK‐242 suppresses the growth, migration and alters susceptibility to ferroptosis in pancreatic cancer cells. (A) Cell viability assay of pancreatic cancer cells treated with TAK‐242 (0, 20 and 40 μM) at 12, 24, 36, and 48 h determined by CCK‐8 (*n* = 5 ***for *p* < 0.001, ****for *p* < 0.0001); (B‐C) Crystal violet staining of cell colonies following TAK‐242 treatment. Quantitative analysis of clonal formation (*n* = 3 **for *p* < 0.01, ****for *p* < 0.0001); (D‐E) Pancreatic cancer cells were treated with TAK‐242 (0, 20 and 40 μM) for 36 h. Crystal violet staining of migration cells were shown (Scale bar: 100 μm). Cell migration rate quantified with ImageJ Plus (*n* = 3, **for *p* < 0.01, ****for *p* < 0.0001); (F) Principal Component Analysis (PCA) of gene expression profiles from TAK‐242–treated and DMSO‐treated PANC‐1 cells. (G) GSEA showing the enrichment of oxidative phosphorylation and inflammatory related pathways; (H) GSEA showing the enrichment of Ferroptosis pathway; (I) The relative level of lipid peroxidation was measured by C11‐BODIPY fluorescence in TAK‐242 or DMSO treated cells; (J) Relative viability of TAK‐242 or DMSO pretreated PANC‐1 cells after 24 h treatment with increasing concentrations of RSL3 (*n* = 3; *for *p* < 0.05).

TAK‐242, a *TLR4*‐specific antagonist, was used to verify the effect of *TLR4* on tumorigenesis in vitro [36, 37]. Cell proliferation assays revealed that TAK‐242 markedly inhibited the growth of pancreatic cell line PANC‐1 and SW1990 cells at both 20 μM and 40 μM concentrations (Figure 7A). Consistently, colony formation assays showed a concentration‐dependent decrease in colony number, indicating that the clonogenic potential of both cell lines was impaired (Figure 7B,C). Transwell migration assays further demonstrated that TAK‐242 significantly suppressed the migratory ability of PANC‐1 and SW1990 cells, with higher concentrations producing stronger inhibition (Figure 7D,E). Together, these results indicated that TAK‐242 inhibited key malignant behaviours of pancreatic cancer cells, including proliferation and migration.

To gain more insights into the impact of TLR4 inhibition on pancreatic cancer cells, we also performed transcriptome sequencing on TAK‐242‐treated PANC‐1 cells. Principal component analysis demonstrated a distinct separation between TAK‐242 and DMSO‐treated groups (Figure 7F). Geneset enrichment analysis revealed that inflammation‐related pathways, including TNFα/NF‐kB and interferon signalling, were significantly suppressed in the TAK‐242‐treated group, consistent with previous findings showing the ability of TLR4 to promote inflammation through the PI3K/AKT‐NF‐kB axis [38] (Figure 7G).

To explore potential vulnerabilities of the TAK‐242‐treated pancreatic cells to various forms of regulated cell death, we evaluated the impact of TAK‐242 on necroptosis, pyroptosis, and ferroptosis pathways. Interestingly, ferroptosis was significantly activated in the TAK‐242–treated group among the list, suggesting the cellular state may have shifted toward a ferroptosis‐prone condition as a result of TAK‐242 treatment (Figure 7H; Figures S7 and S8A–B). To validate this hypothesis, lipid peroxidation was assessed using C11‐BODIPY staining. TAK‐242 treatment led to a pronounced increase in lipid peroxidation levels compared to controls (Figure 7I). Consistently, TAK‐242‐pretreated cells exhibited greater sensitivity to the ferroptosis inducer RSL3, as evidenced by a further reduction in cell viability compared to control cells. Together, these findings suggest that TAK‐242 enhanced the susceptibility of pancreatic cancer cells to ferroptosis (Figure 7J).

## 4 Discussion

Necroptosis, pyroptosis and ferroptosis are forms of regulated cell death closely linked to immune infiltration in the TME, affecting tumour progression and therapeutic response [39, 40]. In this study, after collating a comprehensive set of necroptosis, pyroptosis, and ferroptosis (NPF) related genes, we performed a comprehensive pan‐cancer analysis focusing on exploring their differential expression, prognostic significance, and potential therapeutic targets. The observed variations in gene expression profile and NPF scores among different cancers may stem from intrinsic biological differences inherent to cancer type‐specific microenvironments, differential genetic backgrounds, metabolic alterations, and immune landscapes. Specifically, cancers such as HNSC, COAD, and LUSC exhibiting higher NPF scores likely represent malignancies characterised by strong inflammatory responses and extensive immune infiltration. Conversely, UCS and PCPG cancers showed lower NPF scores, possibly due to a distinct immune surveillance environment or effective activation of anti‐tumour immunity mediated by specific cell death pathways [41, 42, 43]. These findings emphasise that NPF pathways do not play uniform roles across cancers, but rather exhibit context‐dependent functions that can shape the tumour immune landscape. Importantly, such heterogeneity translates into significant prognostic implications, where elevated NPF activity correlates with poor survival in some cancers while indicating favourable outcomes in others. Understanding these patterns can thus provide valuable insights into patient stratification, risk assessment, and the discovery of novel therapeutic targets.

The identification of prognostic genes and the subsequent development of a robust prognostic model further substantiate the clinical relevance of NPF pathways. The distribution of prognostic genes across necroptosis, pyroptosis, and ferroptosis pathways underscores the interconnectedness of these forms of cell death in modulating tumour prognosis and progression. The high predictive accuracy demonstrated by ROC curves validates the potential clinical utility of these pathways as biomarkers for patient stratification and individualised therapeutic strategies. Notably, the close association and functional interplay between pyroptosis and necroptosis in our findings resemble molecular features characteristic of PANoptosis, a recently described integrated form of programmed cell death that incorporates elements of pyroptosis, necroptosis, and apoptosis [44]. Although our study did not explicitly include PANoptosis‐related genes, their inclusion could further elucidate the convergence of these death pathways and offer a more holistic understanding of their roles in cancer. The exclusion of PANoptosis components represents a limitation, and future work incorporating PANoptosis‐specific markers may help refine prognostic modelling and uncover novel therapeutic avenues.

We further evaluated the association between the identified prognostic genes and the tumour immune microenvironment. A significant finding is the identification of TLR4 as a critical molecule associated with tumour progression. Previous studies have indicated that high TLR4 expression in tumour cells may alter the tumour microenvironment through various mechanisms. For instance, activation of TLR4 can promote the secretion of pro‐inflammatory cytokines and chemokines, such as IL‐6, IL‐10, and TNF‐α, which can create an immunosuppressive microenvironment that facilitates tumour growth and metastasis [45, 46]. *TLR4* signalling has also been shown to induce polarisation of tumour‐associated macrophages (TAMs) toward the M2 phenotype, which typically exhibits immunosuppressive, pro‐angiogenic, and tumour‐promoting characteristics [47, 48]. In our study, high *TLR4* expression was significantly associated with increased M2 macrophage infiltration (Rho = 0.74, *p* < 0.001), suggesting that *TLR4* may enhance tumour immune evasion by promoting M2 macrophage infiltration. Furthermore, high *TLR4* expression was positively correlated with CD4+ memory T cell infiltration (Rho = 0.487, *p* < 0.001), which may be related to *TLR4*‐mediated regulation of local pro‐inflammatory cytokine levels that attract and retain CD4+ memory T cells. However, the function of these CD4+ memory T cells in the tumour microenvironment may be impaired, limiting their anti‐tumour activity [49, 50]. In summary, high *TLR4* expression induces the release of pro‐inflammatory factors and alters the immune microenvironment, leading to M2 macrophage polarisation and abnormal CD4+ memory T cell infiltration. Collectively, these findings highlight TLR4's multifaceted role in modulating the immune landscape, promoting tumour immune escape, and advancing cancer progression, thereby reinforcing its potential as a promising target for targeted therapy.

To further validate the therapeutic potential of *TLR4* in cancer, we evaluated the anti‐tumour effects of the specific *TLR4* inhibitor TAK‐242. We found that TAK‐242 significantly inhibited the proliferation, colony formation, and migration of pancreatic cancer cells. This effect is likely mediated by suppression of TLR4‐driven inflammatory signalling. Previous studies have shown that TLR4 promotes tumour progression through activation of pathways such as PI3K/AKT‐NF‐κB in inflammation‐related cancers [38, 51]. Consistently, our transcriptomic analysis revealed that TAK‐242 treatment led to marked downregulation of TNFα/NF‐κB and interferon pathways. It suggested that inhibition of pro‐inflammatory signalling may underlie the observed reduction in tumour cell aggressiveness. In addition to its anti‐proliferative effects, TAK‐242 treatment led to increased lipid peroxidation and enhanced susceptibility to ferroptosis. Transcriptomic analysis revealed a significant upregulation of HMOX1 (Figure S8C). Previous studies have shown that upregulation of HMOX1 can degrade heme and release Fe^2+^ [52]. The increased labile iron can promote lipid peroxidation and make cells more susceptible to ferroptosis. This is consistent with our findings and further underscores the therapeutic potential of TAK‐242.

Finally, although our findings suggest that TAK‐242 affects tumour proliferation, migration, and the susceptibility to ferroptosis, the precise molecular mechanisms remain unclear. Further studies are needed to elucidate specific downstream targets and regulatory mechanisms. Second, our research primarily focused on in vitro experiments, and future studies should evaluate the role of TAK‐242 in in vivo models to better understand its impact on tumour growth and metastasis.

## Author Contributions

**Yu Shen:** conceptualization (lead), investigation (lead), visualization (lead), writing – original draft (lead), writing – review and editing (lead). **Yishu Deng:** investigation (equal), visualization (equal), writing – original draft (equal), writing – review and editing (equal). **Yali Xie:** visualization (equal), writing – review and editing (equal). **Tao Zeng:** writing – review and editing (equal). **Yongjing Liu:** writing – review and editing (equal). **Junwan Lu:** conceptualization (equal), supervision (equal), writing – review and editing (equal). **Jinyan Huang:** conceptualization (equal), funding acquisition (lead), writing – review and editing (lead).

## Conflicts of Interest

The authors declare no conflicts of interest.

## Supporting information


Data S1.

## Data Availability

The raw sequence data reported in this paper have been deposited in the Genome Sequence Archive [53] in National Genomics Data Center [54], China National Center for Bioinformation/Beijing Institute of Genomics, Chinese Academy of Sciences (GSA‐Human: HRA011784) that are publicly accessible at https://ngdc.cncb.ac.cn/gsa‐human. In addition, publicly available datasets used in this study can be accessed from TCGA, GTEx and the GEO projects (GSE71729). The R code and RDS files used in this study have been deposited in a public GitHub repository, https://github.com/SYD0831/Pancancer‐Analysis‐NPF.
