# Supplementary material for: Pan‐Cancer Analyses of Necroptosis, Pyroptosis and Ferroptosis Related Genes Reveal TLR4 as A Potential Therapeutic Target
Source: J Cell Mol Med. 2025 Jul 22;29(14):e70742. doi: 10.1111/jcmm.70742 (PMC12283246; doi:10.1111/jcmm.70742)
Supplement: Supplementary file 1 — Data S1. [file JCMM-29-e70742-s001.docx]

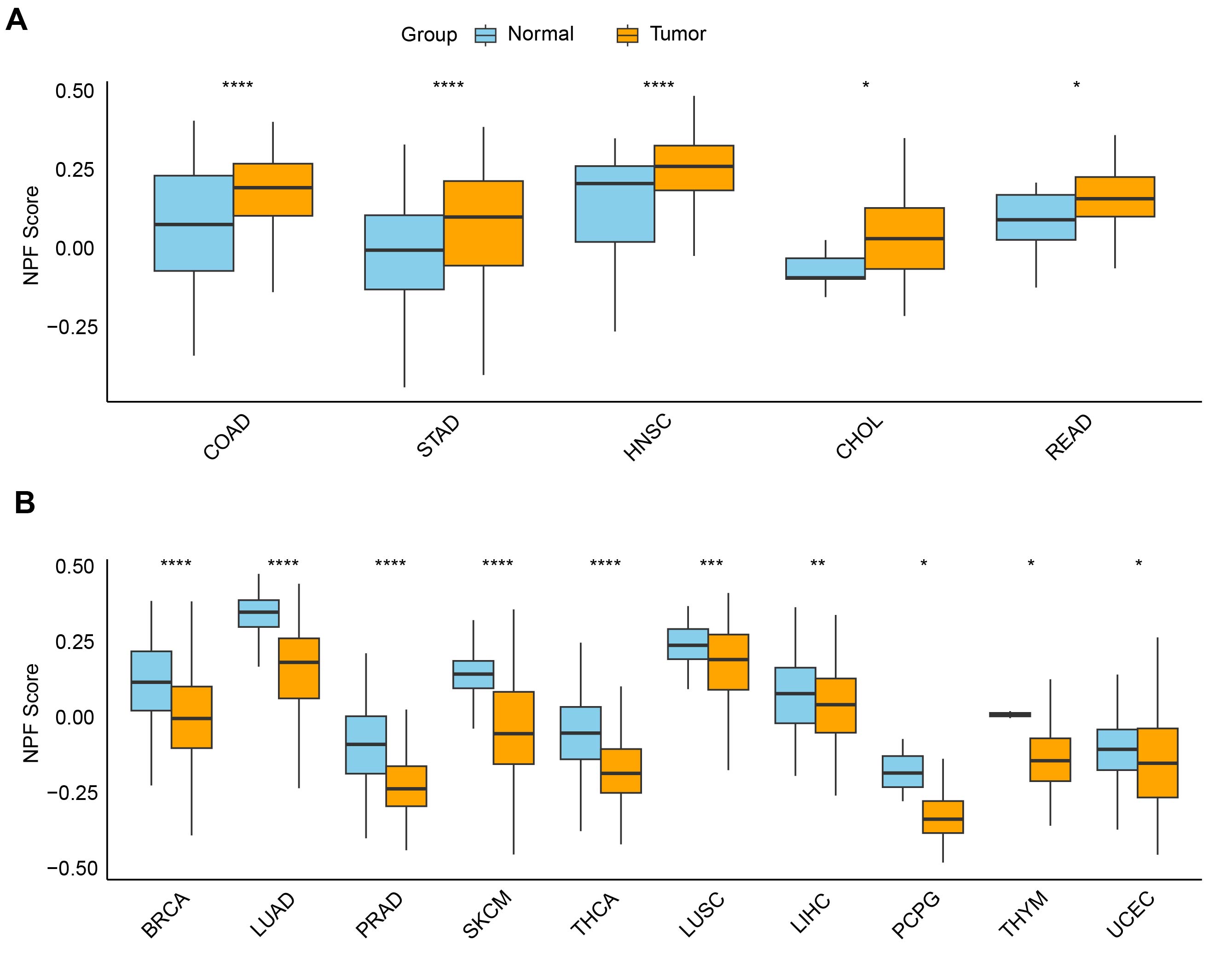


**Figure S1**

**Comparison of NPF Scores Between Tumor and Normal Tissues Across 22 Cancer Types.** (A) Boxplot showing the NPF score between normal tissue and cancer tissue, with the NPF score higher in cancer tissue compared to normal tissue (* *p* < 0.05, *** *p* < 0.001, **** *p* < 0.0001); (B) Boxplot showing the NPF score between normal tissue and cancer tissue, with the NPF score higher in normal tissue compared to cancer tissue(* *p* < 0.05, *** *p* < 0.001, **** *p* < 0.0001).


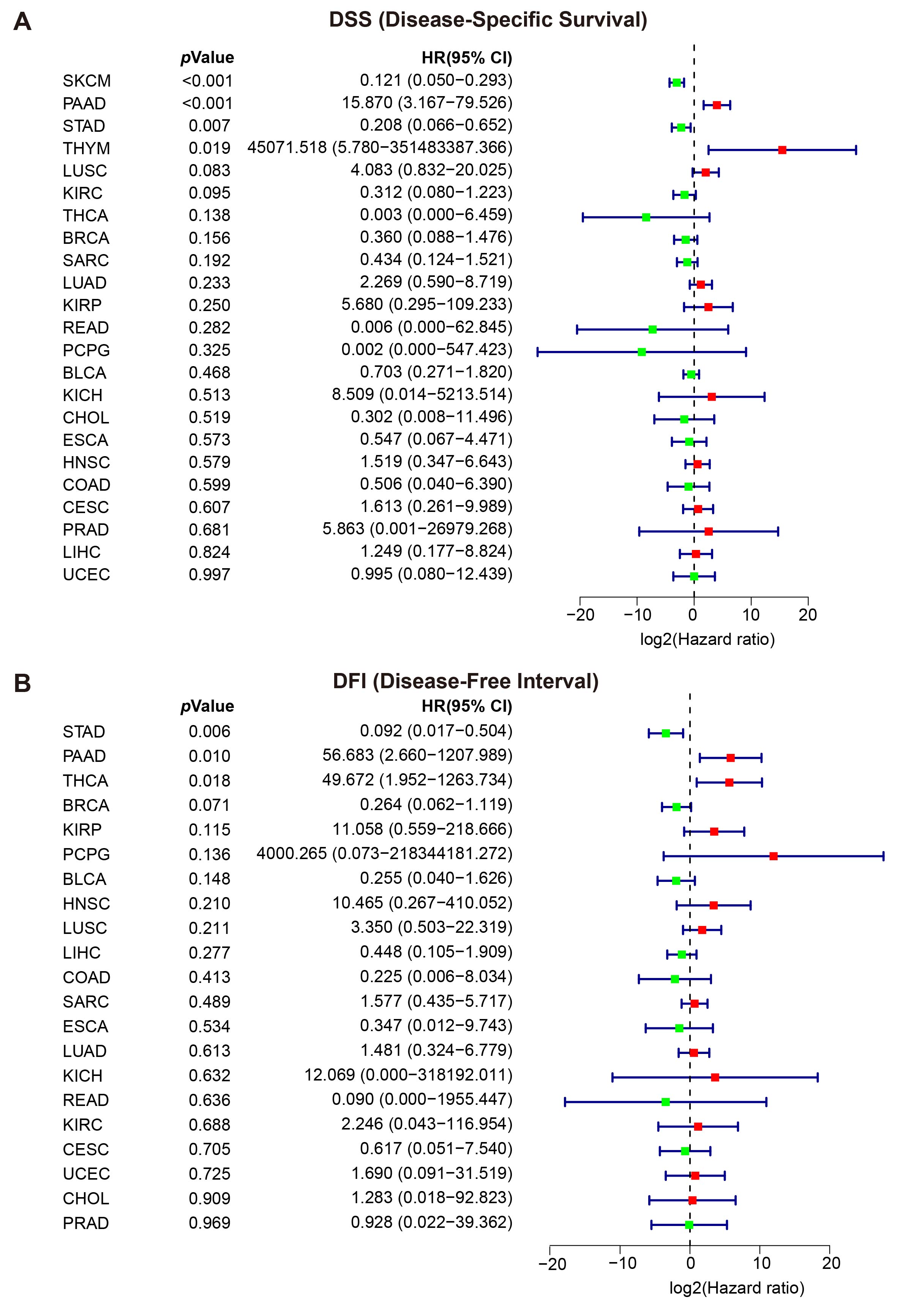


**Figure S2**

**Relationship between the NPF score and clinical data.** (A) Hazard ratio showing that NPF score is associated with DSS in several tumor types; (B) NPF score is associated with DFI in several tumor types. A two-sided, univariate Cox proportional hazards regression analysis was used. HRs with 95% confidence intervals (CIs) are plotted for each tumor type. The color of the forest plot was annotated according to the HR. Red represents NPF score as a risk factor in the indicated cancer type, while Green represents NPF score as a protective factor. Sample sizes: BLCA (n=407), BRCA (n=1098), CESC (n=306), CHOL (n=36), COAD (n=288), ESCA (n=182), HNSC (n=520), KICH (n=66), KIRC (n=531), KIRP (n=289), LIHC (n=371), LUAD (n=515), LUSC (n=498), PAAD (n=179), PCPG (n=182), PRAD (n=496), READ (n=92), SARC (n=262), SKCM (n=469), STAD (n=414), THCA (n=512), THYM (n=119), UCEC (n=181).


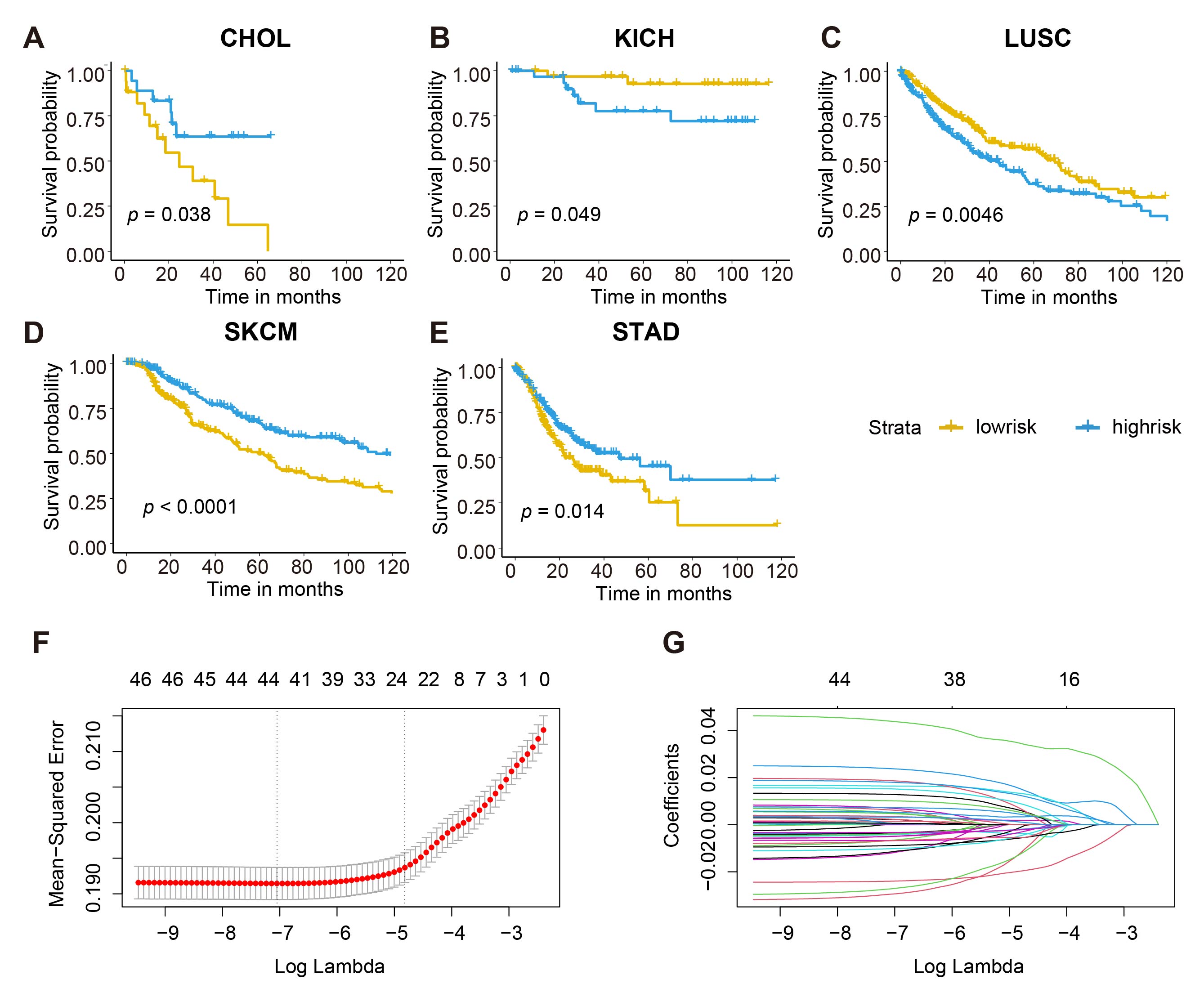


**Figure S3**

**Survival Analysis and Lasso Cox Regression.** (A-E) Survival Curves for High and Low NPF Score Groups in Eight Cancer Types with Significant Differences. A log-rank test was used for statistical analysis; (F) LASSO regression of OS-related NPF genes with significant differential expression; (G) Cross-validation for tuning the parameter selection in the LASSO regression. The optimal parameter value (number of genes = 23) was selected based on the minimum error obtained from cross-validation.


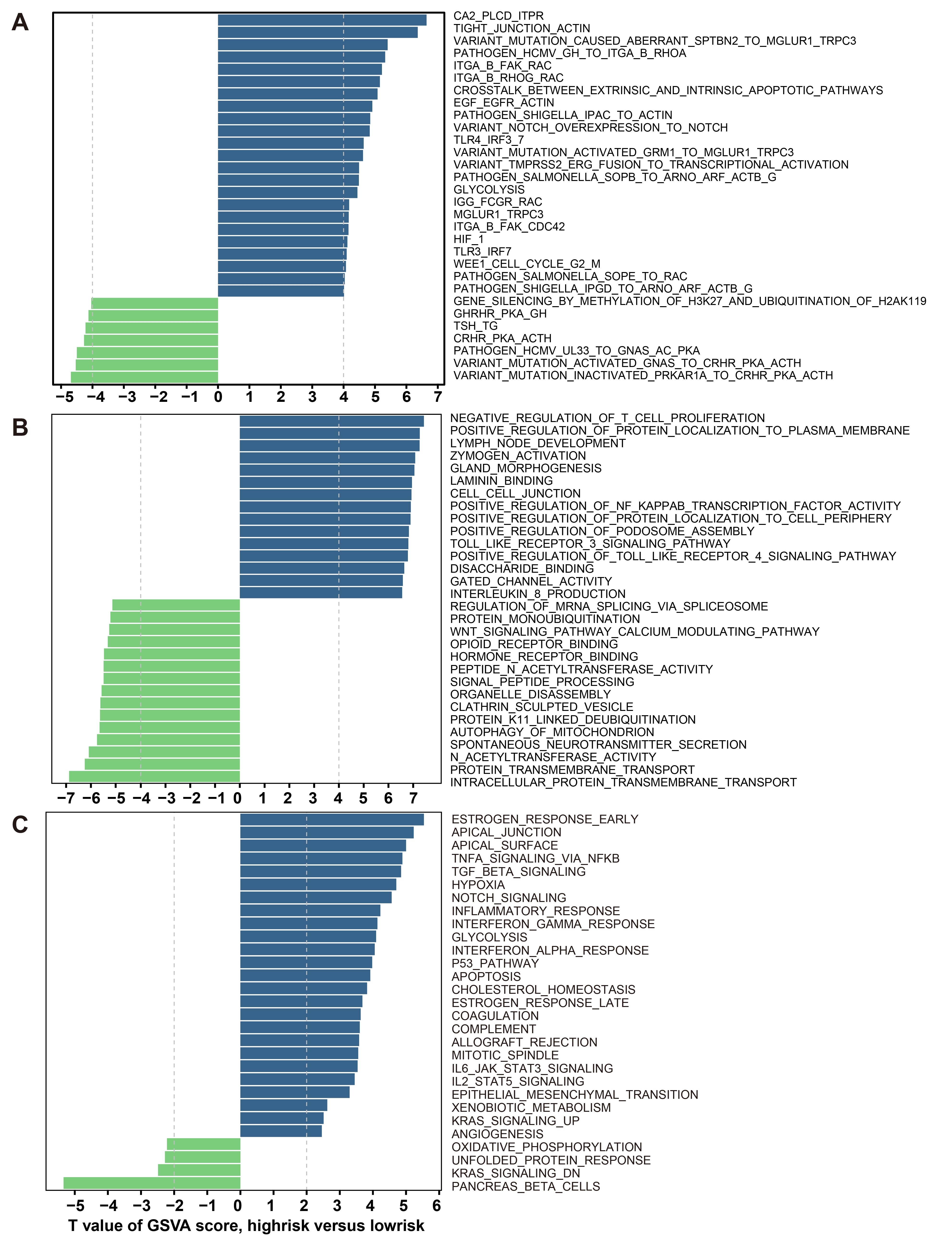


**Figure S4**

**GSVA analysis of High- and Low-Risk Groups in PAAD Patients.** Bar plot showing the top 30 pathways with the highest T-values, comparing enrichment scores between high- and low-risk groups. (A) Result of KEGG medicus gene set; (B) Result of Gene Ontology (GO); (C) Result of hallmark gene set. Blue and green bars indicate pathways enriched in the high-risk and low-risk groups, respectively. Statistical significance was assessed using a two-sided Wilcoxon signed-rank test (*p* < 0.05).

**
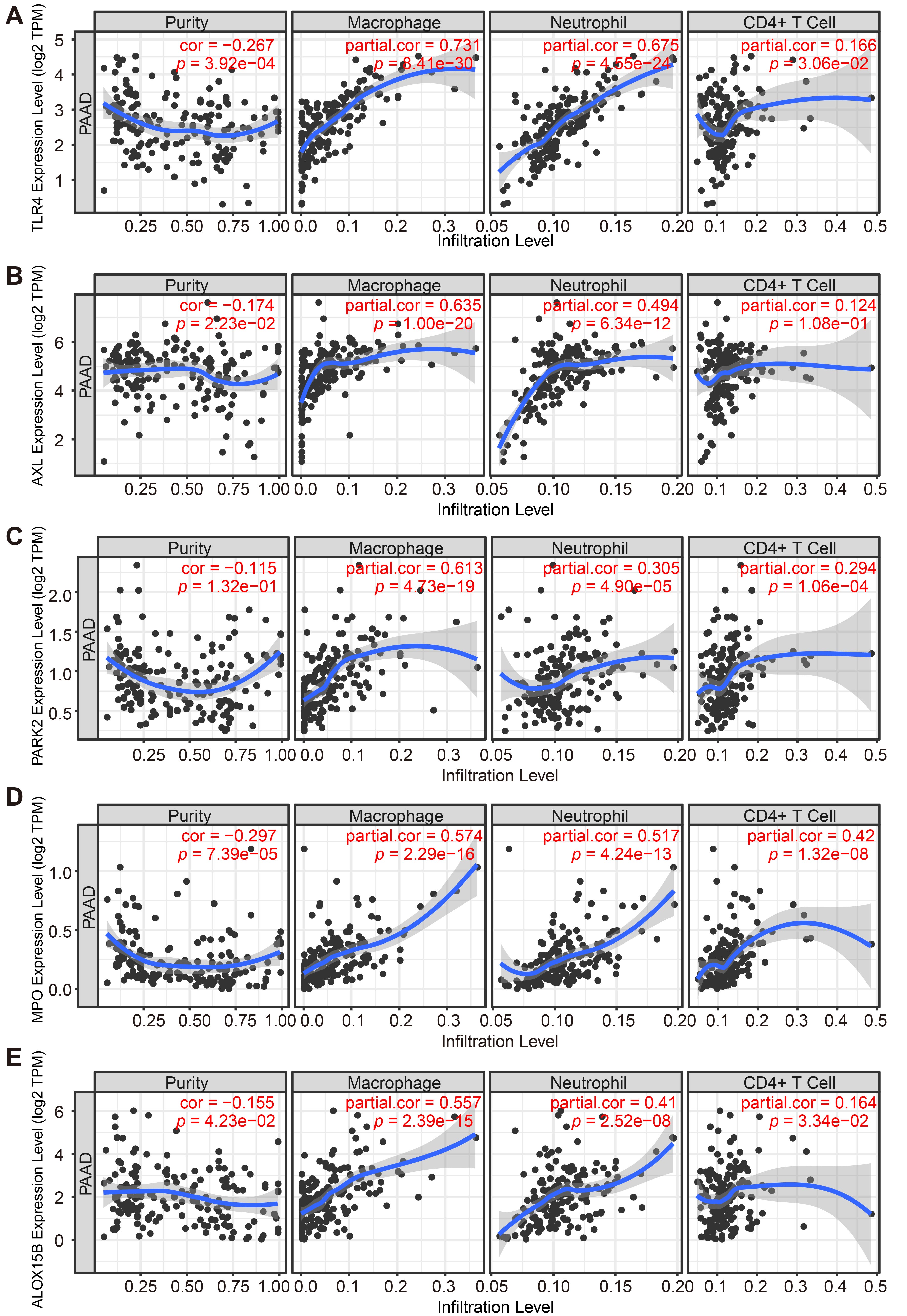
**

**Figure S5**

**Immune Infiltration Analysis (TIMER) of the Top 5 Genes Most Associated with Macrophages Identified by Multivariate Cox Regression.** (A) Correlation between TLR4 expression and immune infiltration levels; (B) Correlation between AXL expression and immune infiltration levels; (C) Correlation between PARK2 expression and immune infiltration levels; (D) Correlation between MPO expression and immune infiltration levels; (E) Correlation between AXL expression and immune infiltration levels. Blue lines represent fitted regression curves with shaded areas indicating confidence intervals. Correlation coefficients (cor) represent the direct linear relationship between gene expression and infiltration levels, while partial correlation coefficients (partial.cor) adjust for tumor purity to account for confounding effects.

**
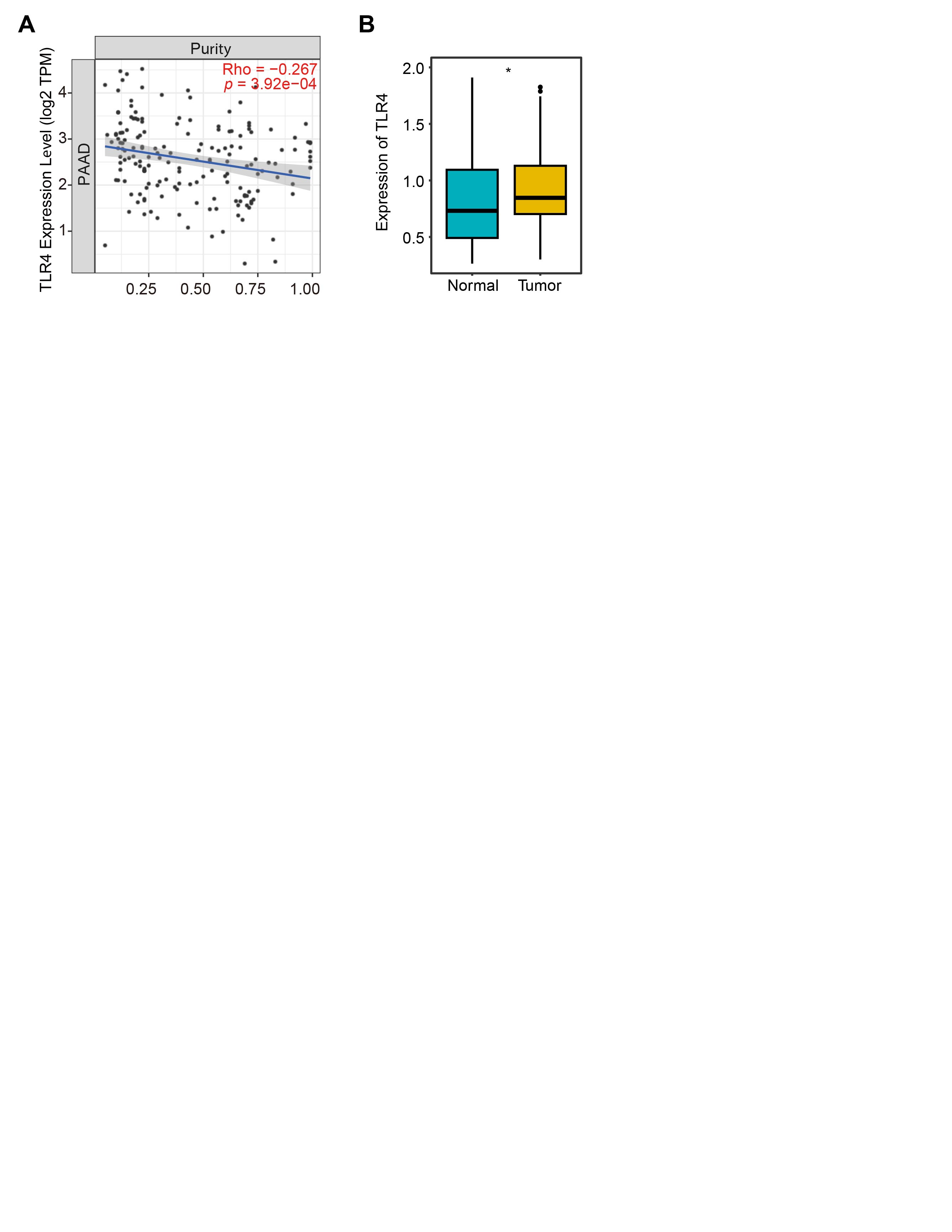
**

**Figure S6**

**Expression profile of TLR4 in pancreatic cancer.** (A) Correlation between TLR4 expression levels and tumor purity in PAAD patients; (B) The expression of TLR4 between normal tissues (n = 46) and PAAD (n = 145) from GSE71729.


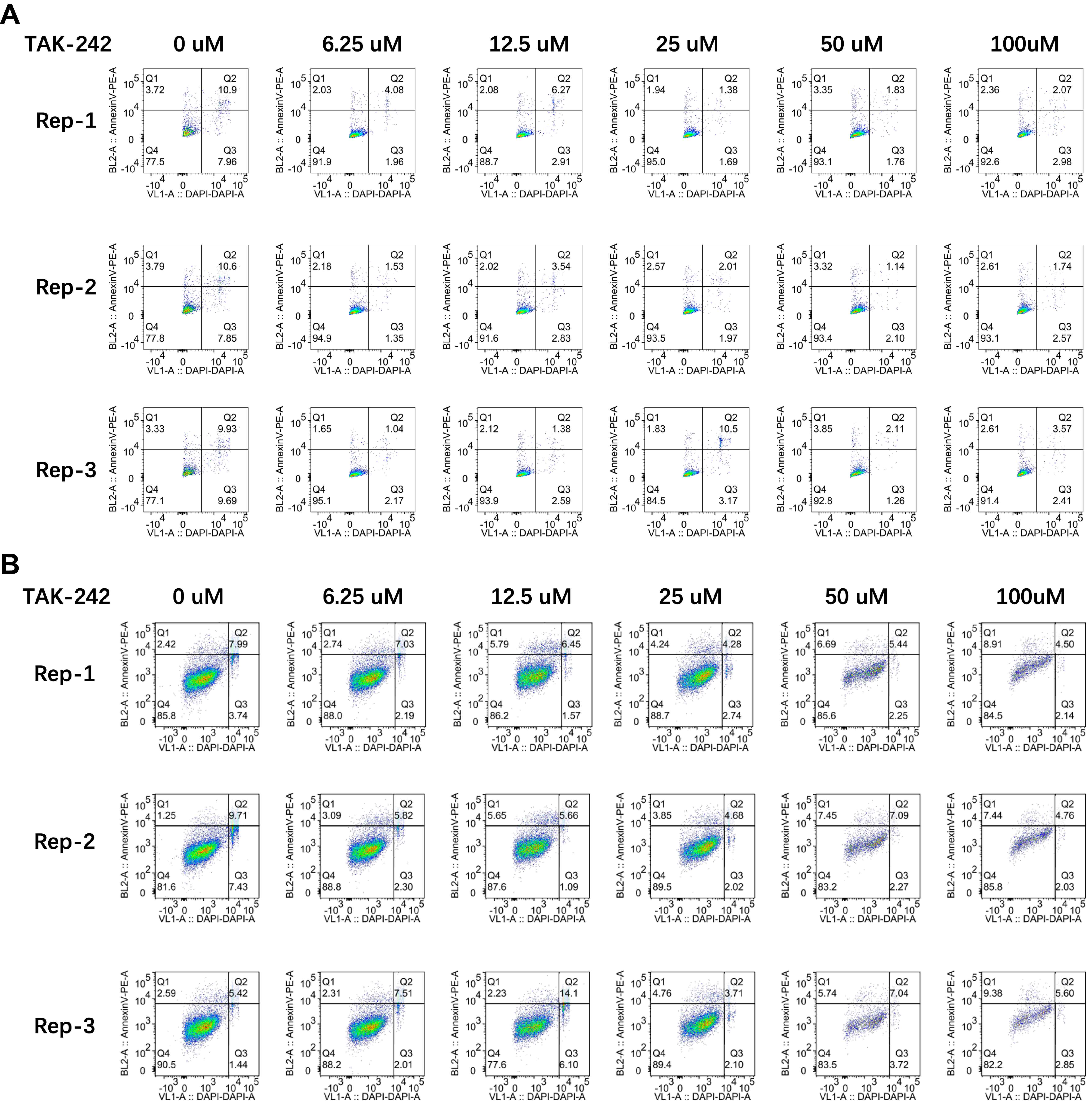


**Figure S7**

**Assessment of cell death in TAK-242–treated PANC-1 cells.** (A) Cell viability assessment at 24 hours following TAK-242 treatment, measured by flow cytometry using Annexin V-FITC and DAPI staining.; (B) Cell viability assessment at 72 hours following TAK-242 treatment, measured by flow cytometry using Annexin V-FITC and DAPI staining.


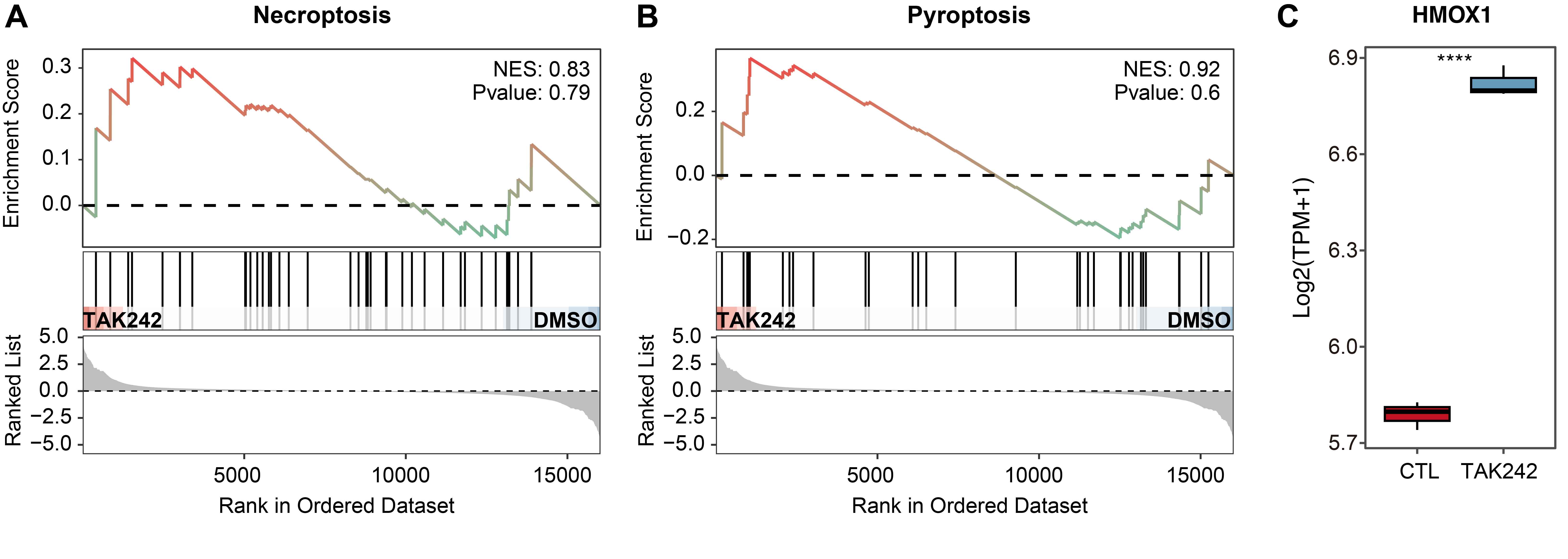


**Figure S8**

**Transcriptomic analysis of TAK-242–treated PANC-1 cells.** (A) GSEA showing the enrichment of Necroptosis pathway; (B) GSEA showing the enrichment of Pyroptosis pathway; (C) Boxplot showing the expression level of HMOX1 (n = 3; ****for *p* < 0.0001).

**Table S1. Necroptosis, pyroptosis and ferroptosis related genes. Bold font is used to highlight column headings.**

| **Necroptosis** | **Reference** | **Pyroptosis** | **Reference** | **Ferroptosis** | **Reference** |
| --- | --- | --- | --- | --- | --- |
| ANAPC11 | PMID:29891719 | AIM2 | PMID:31862870 | ACACA | PMID:35067161 |
| AURKA | PMID:28764929 | BAK1 | R-HSA-5620971 | ACSL1 | PMID:36882396 |
| AXL | PMID:30157175 | BAX | R-HSA-5620971 | ACSL4 | PMID:35180475 |
| BCL2 | PMID:26587781 | CARD8 | PMID:33542150 | AGPS | PMID:38200609 |
| BECN1 | PMID:30057310 | CASP1 | R-HSA-5620971 | AIFM2 | GO:0110075 |
| BIRC2 | PMID:37904685 | CASP3 | R-HSA-5620971 | ALOX12 | PMID:30962574 |
| BIRC3 | PMID:38130918 | CASP4 | R-HSA-5620971 | ALOX12B | PMID:38244223 |
| CASP8 | PMID:31748744 | CASP5 | R-HSA-5620971 | ALOX15 | PMID:36987924 |
| CBL | PMID:34817023 | CASP8 | PMID:34012073 | ALOX15B | PMID:36801644 |
| CDC37 | PMID:25852146 | CD274 | PMID:32929201 | ALOX5 | PMID:37080437 |
| CHUK | PMID:27502281 | CHMP2A | R-HSA-5620971 | ALOXE3 | PMID:34977009 |
| CSNK1A1 | PMID:31932442 | CHMP2B | R-HSA-5620971 | CD36 | PMID:33691090 |
| CYLD | PMID:36035360 | CHMP3 | R-HSA-5620971 | DPP4 | PMID:28813679 |
| FADD | GO:0097527 | CHMP4A | R-HSA-5620971 | FAR1 | PMID:33731874 |
| FAS | GO:0097527 | CHMP4B | R-HSA-5620971 | FASN | PMID:38228715 |
| FASLG | GO:0097527 | CHMP4C | R-HSA-5620971 | FTH1 | GO:0110075 |
| GSK3B | PMID:24984063 | CHMP6 | R-HSA-5620971 | GNPAT | PMID:38041059 |
| HSP90AA1 | PMID:37062702 | CHMP7 | R-HSA-5620971 | GPX4 | GO:0110075 |
| HSPA4 | PMID:38125299 | CYCS | R-HSA-5620971 | HMOX1 | GO:0110075 |
| HSPA8 | PMID:37580406 | CYLD | PMID:31097224 | HSP90 | PMID:37056925 |
| IKBKB | PMID:34363755 | DDX3X | PMID:35692100 | LPCAT3 | PMID:37166352 |
| IKBKE | PMID:34363755 | DPP8 | PMID:29967349 | LTF | PMID:32811647 |
| IKBKG | PMID:34153072 | DPP9 | PMID:29967350 | MPO | PMID:35091012 |
| LRRK2 | PMID:35907404 | EIF2AK2 | PMID:39311631 | NCOA4 | PMID:36752571 |
| MAP3K7 | PMID:36912451 | ELANE | R-HSA-5620971 | NFE2L2 | GO:0110075 |
| MAPKAPK2 | PMID:32366830 | FADD | PMID:31850239 | NOX | PMID:34570416 |
| MERTK | PMID:37403014 | GPX4 | PMID:37272058 | NQO1 | GO:0110075 |
| MIB2 | GO:0010803 | GSDMA | PMID:35110732 | PEX10 | PMID:39097593 |
| MLKL | GO:0097527 | GSDMB | PMID:32299851 | PEX12 | PMID:10562279 |
| MYC | PMID:30209397 | GSDMC | PMID:34012073 | PEX3 | PMID:37488941 |
| NAP1L1 | PMID:32747526 | GSDMD | R-HSA-5620971 | PEX7 | PMID:25261981 |
| OGT | PMID:33231560 | GSDME | R-HSA-5620971 | POR | PMID:33321093 |
| OTULIN | PMID:34625557 | GZMA | PMID:32299851 | SLC11A2 | PMID:36336986 |
| PELI1 | PMID:29078411 | GZMB | R-HSA-5620971 | SLC39A7 | GO:0110075 |
| PPM1B | PMID:25751141 | HMGB1 | R-HSA-5620971 | SLC7A11 | GO:0110075 |
| PRKAA1 | PMID:33779513 | IL18 | R-HSA-5620971 | SQSTM1 | GO:0110075 |
| PRKN | PMID:31681304 | IL1A | R-HSA-5620971 | STEAP3 | PMID:38440354 |
| RBCK1 | PMID:36248876 | IL1B | R-HSA-5620971 | TF | PMID:32203170 |
| RIPK1 | GO:0097527 | IRF1 | R-HSA-5620971 | TFRC | PMID:34011924 |
| RIPK3 | GO:0097527 | IRF2 | R-HSA-5620971 | TMEM164 | GO:0110075 |
| RNF31 | PMID:32944611 | IRF7 | PMID:37930487 | TP53 | PMID:37236507 |
| SHARPIN | PMID:37813853 | MEFV | PMID:37051243 |  |  |
| STUB1 | PMID:26900751 | MIR223 | PMID:37593145 |  |  |
| TANK | PMID:36035360 | NAIP | PMID:37586642 |  |  |
| TBK1 | PMID:37475188 | NEK7 | PMID:31787755 |  |  |
| TICAM1 | PMID:23162759 | NLRC4 | PMID:32295623 |  |  |
| TLR3 | GO:0097527 | NLRP1 | PMID:34719258 |  |  |
| TLR4 | PMID:38367924 | NLRP3 | PMID:35513901 |  |  |
| TNF | GO:0097527 | NLRP6 | PMID:35308537 |  |  |
| TNFAIP3 | PMID:38153253 | NLRP9 | PMID:35090055 |  |  |
| TNFRSF10A | PMID:37443108 | PARK2 | PMID:35143076 |  |  |
| TNFRSF10B | PMID:37446136 | PINK1 | PMID:37788592 |  |  |
| TNFRSF1A | PMID:37615626 | PKM2 | PMID:38342404 |  |  |
| TNFSF10 | PMID:27528614 | PLCG1 | PMID:27464494 |  |  |
| TRADD | PMID:37002200 | PLK4 | PMID:37296437 |  |  |
| TRAF2 | PMID:30988281 | PRF1 | PMID:37221570 |  |  |
| TYRO3 | PMID:31230815 | PYCARD | PMID:31316052 |  |  |
| ZBP1 | PMID:34613770 | RIP1 | PMID:37725754 |  |  |
|  |  | RPTOR | PMID:38010109 |  |  |
|  |  | RRAGA | PMID:37310547 |  |  |
|  |  | RRAGC | PMID:37310547 |  |  |
|  |  | SPATA2 | PMID:37587833 |  |  |
|  |  | TLR4 | PMID:35690810 |  |  |
|  |  | TNF | PMID:36823174 |  |  |
|  |  | TP53 | R-HSA-5620971 |  |  |
|  |  | TP63 | R-HSA-5620971 |  |  |
|  |  | TREM2 | PMID:36068223 |  |  |
|  |  | ZBP1 | PMID:32729116 |  |  |

**Table S2. TCGA Cancer Types and Their Abbreviations.** **Bold font is used to highlight column headings.**

| **TCGA Abbreviation** | **TCGA Full Name** |
| --- | --- |
| BLCA | Bladder Urothelial Carcinoma |
| BRCA | Breast invasive carcinoma |
| CESC | Cervical squamous cell carcinoma and endocervical adenocarcinoma |
| CHOL | Cholangiocarcinoma |
| COAD | Colon adenocarcinoma |
| ESCA | Esophageal carcinoma |
| HNSC | Head and Neck squamous cell carcinoma |
| KICH | Kidney Chromophobe |
| KIRC | Kidney renal clear cell carcinoma |
| KIRP | Kidney renal papillary cell carcinoma |
| LIHC | Liver hepatocellular carcinoma |
| LUAD | Lung adenocarcinoma |
| LUSC | Lung squamous cell carcinoma |
| PAAD | Pancreatic adenocarcinoma |
| PCPG | Pheochromocytoma and Paraganglioma |
| PRAD | Prostate adenocarcinoma |
| READ | Rectum adenocarcinoma |
| SARC | Sarcoma |
| SKCM | Skin Cutaneous Melanoma |
| STAD | Stomach adenocarcinoma |
| THCA | Thyroid carcinoma |
| THYM | Thymoma |
| UCEC | Uterine Corpus Endometrial Carcinoma |

**Table S3. Differentially expressed genes associated with overall survival.**

| ACACA | CHUK | HSP90AA1 | OGT | TF |
| --- | --- | --- | --- | --- |
| ACSL1 | CISD1 | HSPA4 | OTULIN | TFRC |
| ACSL3 | CISD2 | HSPA5 | PARK2 | TICAM1 |
| ACSL4 | CSNK1A1 | HSPA8 | PCBP1 | TLR3 |
| AGPS | CYLD | IKBKB | PELI1 | TLR4 |
| AIFM2 | DDX3X | IKBKE | PEX10 | TNF |
| AIM2 | DHFR | IKBKG | PEX12 | TNFAIP3 |
| AKR1C1 | DHODH | IL18 | PEX3 | TNFRSF10A |
| AKR1C2 | DPP4 | IL1A | PEX7 | TNFRSF10B |
| AKR1C3 | DPP8 | IL1B | PINK1 | TNFRSF1A |
| ALOX12 | DPP9 | IRF1 | PKM2 | TNFSF10 |
| ALOX12B | EIF2AK2 | IRF2 | PLA2G6 | TP53 |
| ALOX15 | ELANE | IRF7 | PLCG1 | TRADD |
| ALOX15B | FADD | LCN2 | PLK4 | TRAF2 |
| ALOX5 | FADD | LPCAT3 | POR | TREM2 |
| ALOXE3 | FAR1 | LRRK2 | PPM1B | TYRO3 |
| ANAPC11 | FAS | LTF | PRF1 | VKORC1L1 |
| AURKA | FASLG | MAP3K7 | PRKAA1 | ZBP1 |
| AXL | FASN | MAPKAPK2 | PRKN |  |
| BCL2 | FDFT1 | MBOAT1 | PROM2 |  |
| BECN1 | FTH1 | MBOAT2 | PYCARD |  |
| BIRC2 | FTL | MEFV | RBCK1 |  |
| BIRC3 | FTMT | MERTK | RIP1 |  |
| CARD8 | GCH1 | MIB2 | RIPK1 |  |
| CASP1 | GCLC | MIR223 | RIPK3 |  |
| CASP3 | GNPAT | MLKL | RNF31 |  |
| CASP4 | GPX4 | MPO | RPTOR |  |
| CASP5 | GPX4 | MYC | RRAGA |  |
| CASP8 | GSDMA | NAIP | RRAGC |  |
| CASP8 | GSDMB | NAP1L1 | SHARPIN |  |
| CBL | GSDMC | NCOA4 | SLC11A2 |  |
| CD274 | GSDMD | NEK7 | SLC3A2 |  |
| CD36 | GSDME | NFE2L2 | SLC40A1 |  |
| CDC37 | GSK3B | NFS1 | SLC7A11 |  |
| CHMP3 | GSS | NLRC4 | SPATA2 |  |
| CHMP4A | GZMA | NLRP1 | SQLE |  |
| CHMP4B | GZMB | NLRP3 | STEAP3 |  |
| CHMP4C | HMGB1 | NLRP6 | STUB1 |  |
| CHMP5 | HMGCR | NLRP9 | TANK |  |
| CHMP6 | HSP90 | NOX | TBK1 |  |
